# Discovering Deleterious Single Nucleotide Polymorphisms of Human AKT1 Oncogene: An In Silico Study

**DOI:** 10.3390/life13071532

**Published:** 2023-07-10

**Authors:** Ruojun Zhang, Nahid Akhtar, Atif Khurshid Wani, Khalid Raza, Vikas Kaushik

**Affiliations:** 1 School of Life Sciences and Technology, Tongji University, Shanghai 200092, China; 2 School of Bioengineering and Biosciences, Lovely Professional University, Phagwara 144411, India; 3 Department of Computer Science, Jamia Millia Islamia, New Delhi 110025, India

**Keywords:** SNPs, oncogene, AKT1, kinase

## Abstract

Background: AKT1 is a serine/threonine kinase necessary for the mediation of apoptosis, angiogenesis, metabolism, and cell proliferation in both normal and cancerous cells. The mutations in the AKT1 gene have been associated with different types of cancer. Further, the AKT1 gene mutations are also reported to be associated with other diseases such as Proteus syndrome and Cowden syndromes. Hence, this study aims to identify the deleterious AKT1 missense SNPs and predict their effect on the function and structure of the AKT1 protein using various computational tools. Methods: Extensive in silico approaches were applied to identify deleterious SNPs of the human AKT1 gene and assessment of their impact on the function and structure of the AKT1 protein. The association of these highly deleterious missense SNPs with different forms of cancers was also analyzed. The in silico approach can help in reducing the cost and time required to identify SNPs associated with diseases. Results: In this study, 12 highly deleterious SNPs were identified which could affect the structure and function of the AKT1 protein. Out of the 12, four SNPs—namely, G157R, G159V, G336D, and H265Y—were predicted to be located at highly conserved residues. G157R could affect the ligand binding to the AKT1 protein. Another highly deleterious SNP, R273Q, was predicted to be associated with liver cancer. Conclusions: This study can be useful for pharmacogenomics, molecular diagnosis of diseases, and developing inhibitors of the AKT1 oncogene.

## 1. Introduction

In-depth insight into genetic variation has emerged as a capacious challenge in the field of genomic research, owing to the presence of a large number of genetic variations in the genome of humans. Assessment of the variations in the genome could serve as a foundation stone to obtain better insight and understand the role of these variations in disease susceptibility, as well as their response to personalized treatment [1]. Additionally, the progressive development in the field of epigenetics and genomics has enabled the gathering of more information and paved the way for new avenues of research for assessing, understanding, and elucidating the mechanisms of genetic disorders and defects [2].

In general, a single nucleotide polymorphism (SNP) is an alteration of a single nucleotide, i.e., adenine (A)/cytosine (C)/guanine (G)/thymine (T) at the genetic (DNA) level. Recently, a type of SNP called non-synonymous SNP (nsSNP) has gained the interest of researchers as it could serve as a potential biomarker for the diagnosis of various diseases. These nsSNPs are responsible for the substitution of amino acids within the sequence of the protein, which further imposes a negative effect on physiochemical properties of natural proteins such as dynamics, protein–protein interaction, and stability [3,4]. Therefore, it is a matter of concern as nsSNPs in genes associated with apoptosis, cell cycle regulation, and differentiation may exhibit deleterious outcomes [5]. One such gene is AKT1, which encodes for RAC-alpha serine/threonine-protein kinase, which is located in chromosome 14 (14q32.33) [6]. As per the annotation release 109.20210514, GRCh38.p13 (*Homo sapiens*), the molecular location of this gene spans from 104,769,349 to 104,795,748 base pairs (bp) on chr14 (NCBI). This gene translates to one out of three members of the human AKT serine/threonine protein kinase family, which are usually stated as protein kinase B alpha (α), beta (β), and gamma (γ). These highly identical AKT proteins encompass the C-terminal regulatory domain, N-terminal pleckstrin homology domain, and serine/threonine-specific kinase domain. Moreover, the protein encoded by this gene becomes phosphorylated via phosphoinositide 3-kinase (PI3K) [7]. Furthermore, AKT/PI3K serves as a vital component in numerous signaling pathways involving ligands (membrane-bound) such as G-protein coupled receptors (GPCR), integrin-linked kinase (ILK), and receptor tyrosine kinases (RTK). Additionally, it also regulates various cellular functions such as angiogenesis (in both malignant and normal cells), cell multiplication, metabolism, and survival [8].

An extensive investigation of this gene has unveiled that AKT is an essential signaling hub that involves interaction with more than 100 downstream target substrates and is involved in tumor cell survival. This occurs as survival factors suppress the apoptosis through a transcription-independent manner by activating AKT1 which later becomes phosphorylated and inactivates the function of machinery responsible for apoptosis [9,10,11]. Additionally, proteins of AKT are also involved in the mammalian target of the rapamycin (mTOR) signaling pathway which regulates the eukaryotic translation initiation factor 4F (eIF4E) complex assembly, where this pathway is involved in the stimulation of extracellular signals from cytokines and growth factors which are dysregulated in numerous cancers [12]. Moreover, a mutation in this gene is associated with excessive tissue proliferation and different types of cancers (breast cancer, colorectal cancer, and ovarian cancer) [13]. AKT also participates in maneuvering glucose uptake via mediating insulin-induced translocation of the GLUT4/SLC2A4 glucose transporter to the cell surface [14,15].

Over the past decades, various in silico approaches have been developed for screening, identifying, and predicting the function of SNPs and the effect of deletion of nsSNPs in the candidate protein. Based on in silico approaches, the functional disease susceptible nsSNPs in various genes such as DEFB1, LEP, AGT, EPHX1, GJA3, GSTM1, HLA-G, GSTP1, TYR, and GSTT1 have been identified successfully [16,17,18,19,20,21,22,23]. This work aims to identify highly deleterious missense SNPs of the ATK1 gene via several in silico analyses. Further, in silico analyses were performed to assess the impact of these highly deleterious missense SNPs on the functionality and stability of the mutant RAC-alpha serine/threonine-protein kinase protein. This study also aims to determine the association of these highly deleterious AKT1 missense SNPs with different cancers. This study can be useful for pharmacogenomics, molecular diagnosis of diseases, and developing inhibitors of the AKT1 oncogene [24,25].

## 2. Materials and Method

**Figure 1:**
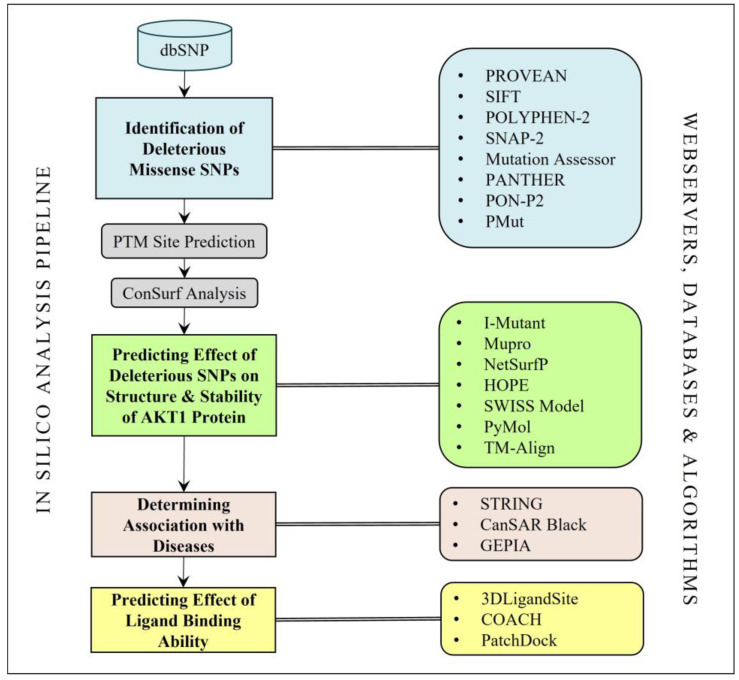
The methodology used for the analysis of the AKT1 missense SNPs.

The schematic representation of the in silico analysis performed in this study is shown in Figure 1.

### 2.1. Single Nucleotide Polymorphism Dataset Retrieval

The dataset for the SNPs present in the human AKT1 gene was retrieved from the dbSNP database (https://www.ncbi.nlm.nih.gov/snp/, accessed on 15 July 2021). The dbSNP is an archive of several SNPs, microsatellites, and deletion insertion polymorphisms which can be used for a wide range of biological research, such as association studies, pharmacogenomics, evolutionary studies, and functional analysis [26]. The amino acid sequence information of the RAC-alpha/threonine-protein kinase protein encoded by the human AKT1gene was obtained from the UniProtKB database with UniProt ID: P31749.

### 2.2. In Silico Identification of the Deleterious Human AKT1 Missense SNPs

It is important to differentiate between benign and deleterious SNPs. For the analysis of the pathogenic nature of SNPs, several webservers and algorithms are available. In this study, 8 different webservers: PROVEAN, SIFT, POLYPHEN-2, SNAP-2, Mutation Assessor, PANTHER, PON-P2, and P-MUT were used to identify the deleterious nsSNPs which can be further subjected to downstream analysis. The use of these different algorithms can be helpful in the prediction of harmful missense SNPs with improved reliability [27].

(a) PROVEAN (Protein Variation Effector Analyzer) webserver version 1.1 was used (http://provean.jcvi.org/index.php, accessed on 17 July 2021). This webserver helps to predict if the single or multiple amino acid substitutions/deletions/insertions will have any effect on protein function [28]. PROVEAN uses an alignment-based score named the delta alignment score (DAS) to predict the effects of the nsSNPs as either deleterious or neutral [28]. The SNPs which have DAS ≤ −2.5 are classified as deleterious and the SNPs above DAS-2.5 are classified as neutral [28]. The list of coordinates and variants was provided as input to the server using default parameters.
(b) SIFT (Sorting Intolerant from Tolerant) (https://sift.bii.a-star.edu.sg/www/Extended_SIFT_chr_coords_submit.html, accessed on 17 July 2021) helps to filter a multitude of SNPs using the sequence homology method based on the assumption that the amino acid substitutions in the evolutionarily conserved regions have a higher probability of affecting the protein function [29]. The SIFT algorithm classifies the nsSNPs into tolerant or deleterious based on the SIFT score which ranges between 0 and 1 [29]. The nsSNPs with a SIFT score below 0.05 are considered deleterious while nsSNPs with a SIFT score ≥ 0.05 are considered tolerant [29]. The list of chromosome coordinates, orientation, and alleles was provided as input to the server.
(c) PolyPhen-2 (Polymorphism phenotyping version 2) classifies the SNPs into three categories: benign, possibly damaging, and probably damaging based on the probabilistic score, ranging between 0 and 1, determined using the machine learning method (http://genetics.bwh.harvard.edu/pph2/, accessed on 18 July 2021) [30]. This server uses an algorithm that selects three structure-based and eight sequence-based predictive features to classify the SNPs [31]. The protein sequence information in FASTA format along with the information related to the substitution and its position were submitted as input to the server.
(d) SNAP-2 was another webserver used to analyze the functional effects of the nsSNPs (https://rostlab.org/services/snap2web/, accessed on 18 July 2021). The amino acid sequence of the AKT1 protein in FASTA format was used as input. This tool uses a neural network approach to classify variants as either effective or neutral on the basis of the SNAP-2 score which is calculated by evaluating various sequence and variant features [32]. The SNAP-2 score between −100 and 0 implies the variants are neutral while the SNAP-2 score ranging from 0 to 100 implies the SNPs will affect the protein function [32].
(e) Mutation Assessor webserver (http://mutationassessor.org/r3/, accessed on 19 July 2021) uses evolutionary conservation patterns to determine the functional impact score which is then used to classify the amino acid substitutions as having a low, medium, or neutral functional impact [33]. The UniProt protein accession of the protein and amino acid substitution information were provided as input.
(f) PANTHER (http://pantherdb.org/tools/csnpScore.do, accessed on 19 July 2021) (Protein analysis through evolutionary relationship) webserver takes the input as the protein sequence in FASTA format and substitution information in the standard amino acid substitution format. It uses the position-specific evolutionary preservation (PSEP) method to predict the causal role of the nsSNPs in human diseases by classifying the nsSNPs as probably benign or probably damaging (Tang et al., 2016).
(g) PON-P2 (http://structure.bmc.lu.se/PON-P2/, accessed on 20 July, 2021) webserver takes the UniProt ID of the protein along with the substitution information as inputs. It uses a machine learning-based method trained using the VariBench database to classify the amino acid substitutions as neutral, pathogenic, or unknown [34].
(h) PMut (http://mmb.irbbarcelona.org/PMut/analyses/new/, accessed on 21 July 2021) webserver uses a neural network-based method trained using the SwissProt dataset to determine the pathological nature of SNPs and classify them as disease-causing or neutral [35].

### 2.3. Post-Translational Modification Sites Prediction

For the identification of the post-translational modification (PTM) sites in the AKT1 protein, the MusiteDeep webserver was used [36]. The amino acid sequence of the AKT1 protein was provided in FASTA format as input to the webserver. It uses deep learning methods to predict and visualize the post-translational modification sites in proteins [36].

### 2.4. In Silico Prediction of the Evolutionarily Conserved Residues in Human AKT1 Protein

The conserved amino acids in the human AKT1 protein were predicted using the ConSurf webserver (https://consurf.tau.ac.il/, accessed on 22 July 2021). It uses either the maximum likelihood method or empirical Bayesian inference to analyze the evolutionary conservation of amino acids in proteins which can be useful in determining the regions which can be vital for protein structure and function [37,38]. The protein sequence of the AKT1 protein was provided in FASTA format as input and then ConSurf analysis was conducted by applying CSI-BLAST against the UNIREF-90 protein database to search for homologs and choosing the recommended parameters. The server classifies the residues based on a color-coded conservation score (CS) ranging between 1 and 9 into three groups: variable (CS = 1 to 4), average (CS = 5), and conserved (CS = 6 to 9) [37].

### 2.5. Predicting the Effects of the Most Deleterious SNPs on the Stability of AKT1 Protein

For predicting the effects of most deleterious nsSNPs on the stability of the AKT1protein, two webservers, I-Mutant and MUpro, were used (http://gpcr2.biocomp.unibo.it/cgi/predictors/I-Mutant3.0/I-Mutant3.0.cgi, accessed on 22 July 2021 and http://mupro.proteomics.ics.uci.edu/, accessed on 22 July 2021). I-Mutant uses a support vector machine (SVM)-based method to predict the protein stability due to single point mutations [39]. The protein sequence of the AKT1 protein in one letter residue code, position of the mutation, and mutated residue symbol in one letter code was provided as input to the I-Mutant server. On the basis of the predicted free energy change (DDG) value, the webserver classifies the effects of the mutants on protein stability as a large decrease in stability (DDG: <−0.5), large increase in stability (DDG: >0.5), or a neutral stability $\left(-0.5\leq \mathrm{DDG}\geq 0.5\right)$. For the MUpro webserver, the protein sequence of AKT1 protein in one letter residue code, the position of the mutation, original amino acid, and mutated amino acid symbol in one letter code was also provided as input. MUpro also uses the SVM method to predict the change in protein stability due to single-site mutations [40]. Similar to the I-Mutant webserver, MUpro also predicts the increase or decrease in stability by considering the DDG value [40].

### 2.6. Solvent Accessible Area Prediction Using NetSurfP

For the prediction of surface accessible amino acids in the AKT1 protein sequence, the NetSurfP 3.0 webserver available at https://services.healthtech.dtu.dk/services/NetSurfP-3.0/ was used (accessed on 23 May 2023). The AKT1 protein sequence in FASTA format was provided as input. This server uses an artificial neural network trained using various experimentally determined protein structures to determine whether the amino acids are buried or exposed residues [41].

### 2.7. Predicting the Effects of the Most Deleterious SNPs on the Structure of AKT1 Protein

The effects of the most deleterious SNPs on the 3D structure of AKT1 protein were predicted by Project HOPE (Have your Protein Explained webserver available at https://www3.cmbi.umcn.nl/hope/, accessed on 25 July 2021). The UniProt accession code of the AKT1 protein and mutation information were provided as input. The HOPE webserver collects and combines data from various databases such as UniProt, and other web services (Venselaar et al., 2010). The HOPE webserver provides information about the schematic structure of wild and mutant residues, the difference in the properties of the mutated and wild-type amino acids, the impact of the different properties of wild and mutant residues on the flexibility and interactions of the proteins along with several other pieces of information, figures, and animations [42].

### 2.8. Protein–Protein Interaction Analysis Using STRING

The STRING database (Search Tool for the Retrieval of Interacting Genes/Proteins) was used to determine the AKT1 protein association network. The STRING database is available at https://string-db.org/ accessed on 31 August 2021. The UniProt protein identifier of the AKT1 protein (P31749) was provided as input and *Homo sapiens* was selected as the organism. The STRING server predicts the protein–protein interactions on the basis of information available from data mining, different experiments, and databases [43]. The protein association network images are generated using the spring model.

### 2.9. Determining the Highly Deleterious AKT1 Missense SNPs Association with Cancer

The association of highly deleterious AKT1 missense SNPs with cancer was predicted by searching the mutation profile generated with the CanSAR Black database available at https://cansarblack.icr.ac.uk/ (accessed on 31 August 2021). The database was searched using the UniProt protein identifier of the AKT1 protein (P31749).

### 2.10. Analysis of AKT1 Gene Expression in Liver Hepatocellular Carcinoma and Survival Rate

As the substitution R273Q was found to be associated with liver cancer, the effect of AKT1 gene expression in liver cancer was analyzed. Furthermore, the effect of the overexpression or low expression of the AKT1 gene in liver cancer patients was also evaluated. For the analysis of gene expression and survival rate, the Gene Expression Profiling Interactive Analysis (GEPIA) webserver’s boxplot and survival analysis tools were used (http://gepia.cancer-pku.cn/index.html, accessed on 31 August 2021). It provides information about tumor/normal differential expression analysis, patient survival analysis, and other information by analyzing RNA sequencing data from different sources such as the Cancer Genome Atlas (TCGA) and the Genotype-Tissue Expression projects [44]. For this analysis, gene name: AKT1 and cancer type: LIHC (liver hepatocellular carcinoma) were used as input queries. For gene expression analysis, the boxplot tool was used. In survival analysis, the cases were divided into two groups: low expression and high expression based on the median expression level of the AKT1 gene.

### 2.11. Prediction of 3D Model of the Mutant Proteins

The mutations at the conserved positions are more likely to be damaging the structure and function of proteins. Hence, the effect of mutations at 100% conserved residues on the structure of the AKT1 protein was studied. Altogether, 4 mutations—G157R, G159V, G336D, and H265Y—were at 100% conserved positions. These four SNPs were also predicted as highly conserved (ConSurf score: 9) using the ConSurf tool for comparison of the structure of wild-type protein and mutant proteins, and the 3D structures of mutant proteins were generated using the SWISS-MODEL online server available at https://swissmodel.expasy.org/ accessed on 31 August 2021. PDB ID: 3O96 (RAC-alpha serine/threonine-protein kinase Crystal Structure of Human AKT1 with an Allosteric Inhibitor) was used as a template for modeling all the mutant proteins and it has 99.77% sequence similarity with the query protein. Mutant proteins were modeled by manually replacing the mutant residue at the related position of the wild-type protein’s FASTA file.

### 2.12. Wild and Mutant AKT1 Proteins Structure Comparison

First of all, the 3D structure of the wild AKT1 protein and the mutants of AKT1 proteins were compared using PyMOL software. PyMOL software was used to identify and compare different types of interactions among the residues in the wild and the selected mutant proteins. The bonds and interaction between the residues are important for the structure and function of proteins. After comparing the structures with PyMOL software, the structures were further compared by aligning the protein structures. The protein structure alignment to determine the structural deviation of superimposed wild and mutant AKT1 proteins was determined using the TM-align webserver. (https://zhanglab.ccmb.med.umich.edu/TM-align/, accessed on 31 August 2021). This TM-align algorithm generates a RMSD value and TM-score (ranges from 0 to 1) which can be used to determine the structural similarity between superimposed proteins [45]. The TM-score of 1 implies a perfect match between the superimposed protein structures. Furthermore, the TM-score below 0.3 implies the structural similarity is random and the TM-score between 0.5 and 1 denotes the structures are in the same fold. Furthermore, the higher RMSD value suggests higher structural dissimilarity.

### 2.13. Effect of Point Mutations on AKT1 Protein Function

The FASTA sequence of the AKT1 protein was deployed to check for the effect of point mutations. We deployed R software (https://www.r-project.org/, accessed on 23 January 2022) and package protr [46].

### 2.14. Prediction of AKT1 Protein–Ligand Binding Sites

The ligand binding sites on the AKT1 protein were predicted using COACH (https://zhanggroup.org/COACH/, accessed on 2 September 2021) and 3DLigandSite (http://www.sbg.bio.ic.ac.uk/3dligandsite/, accessed on 2 September 2021) [47,48]. The COACH webserver uses the combination of two methods, TM-SITE and S-SITE, using a support vector machine to predict the ligand binding site prediction [47]. The 3DLigandSite webserver predicts the ligand binding sites by superimposing the ligands from similar structures on the model of the target protein [48]. In both, the webservers’ protein sequence in FASTA format was provided as input.

### 2.15. Molecular Docking Analysis

Further study to analyze the effect of SNPs on the interaction between ligand and wild-type and mutant proteins was undertaken by performing a molecular docking study using the PatchDock webserver available at the website: https://bioinfo3d.cs.tau.ac.il/PatchDock/php.php accessed on 5 September 2021 [49]. The PatchDock server is a geometry-based molecular docking algorithm and determines the ligand–receptor interaction based on molecular shape complementarity and atomic contact energy [49]. For molecular docking, the protein structures of wild-type and mutant proteins were used as receptors. 1-(1-(4-(7-phenyl-1H-imidazo[4,5-g] 7midazole7e-6-yl) benzyl) piperidin-4-yl)-1H-benzo[d]7midazole-2(3H)-one, which is also known as AKT inhibitor VIII, was used as the ligand. AKT inhibitor VIII is an allosteric inhibitor of the AKT1 protein [50]. The 3D conformer of the AKT inhibitor VIII was obtained from PubChem with PubChem ID: 135398501 in SDF format which was then converted to PDB format using Open Babel software [51]. While docking, protein–small ligand docking was selected on the server and an RMSD value of 1.5 Å was used. After docking, the complexes were refined using a FireDock webserver [52].

### 2.16. Analysis of the Effect of 3′UTR SNPs on microRNA (miRNA) Binding Sites

The association between miRNA and SNPs was evaluated using the PolymiRTS (polymorphisms in miRNAs and their target sites) database [53]. The PolymiRTS database consolidates information from hybrid sequences, crosslink experiments, and miRNA ligation interactions. It can determine the position of non-coding SNPs, whether they occur in miRNA target sites or the seed regions of miRNA. Additionally, it assesses the functional impacts of SNPs in miRNA target sites and seed regions. In this study, the gene symbol AKT1 was submitted to the PolymiRTS database to analyze the consequences of non-coding SNPs. The effects of these variants are categorized as follows: “C” (the derived allele creates a new miRNA site), “D” (the derived allele disrupts a conserved miRNA site), “N” (the derived allele disrupts a non-conserved miRNA site), and “O” (it is not possible to determine the ancestral allele).

### 2.17. Analysis of Association of AKT1 SNPs with Different Cancers

The data from The Cancer Genome Atlas program (TCGA) available at the Genomic Data Commons (GDC) Data Portal (https://portal.gdc.cancer.gov/ accessed on 9 June 2023) was explored for analyzing the AKT1 SNP association with different cancers. In the GDC portal, the gene name AKT1 was searched under the “Gene” option of the “Exploration” page. Then, the mutations option was clicked to see different mutations in AKT1 that have been reported to be associated with cancer.

## 3. Results

### 3.1. Single Nucleotide Polymorphism Dataset Retrieval

Altogether, 12,417 SNPs were identified for the AKT1 gene from the dbSNP database where 10,253 were present in the intron region, 246 were synonymous, 293 were missense (Supplementary Table S1), and the rest were in-frame deletion, in-frame insertion, and non-coding transcript variant.

### 3.2. In Silico Identification of the Deleterious Human AKT1 Missense SNPs

The 293 missense AKT1 SNPs were analyzed by eight different webservers to predict the SNPs which can have an effect on the AKT1 function. The analysis of these SNPs using different webservers can be beneficial in predicting those missense SNPs that can damage AKT1 functions. The results of missense AKT1 SNPs analysis using different webservers are shown in Supplementary Table S2. After the analysis of 293 missense AKT1 SNPs, 12 SNPs were predicted to be deleterious by all the webservers unanimously. Hence, these 12 missense AKT1 SNPs (Table 1) were selected for further analysis of their effect on the structure and function of the AKT1 protein.

### 3.3. Post-Translational Modification Sites Prediction

PTM involves the addition of different functional groups to the side chain of a protein’s amino acid residues [54]. These PTMs are necessary for the regulation of protein–protein interactions, protein folding, and other protein functions [27,36]. They also play an important role in disease prognosis [55,56]. Hence, it is important to identify the PTMs in the study of diseases. Among the missense SNPs which were predicted as deleterious by all the webservers used in this study, the PTM site for phosphorylation and glycosylation was identified at Thr312 residue. The MusiteDeep webserver did not predict PTM for the remaining SNP variants.

### 3.4. In Silico Prediction of the Evolutionarily Conserved Residues in Human AKT1 Protein

According to the ConSurf analysis, all the selected deleterious missense AKT1 SNPs were located in the conserved regions of the AKT1 protein. The SNPs G157R, G159V, F217L, G336D, A391G, H265Y, R273Q, L321P, G345S, T312I, and G286R were located at residues with a conservation score of 9 and the remaining mutants (His405Pro) were located at residues with conservation score of 8. Furthermore, the neural network algorithm of ConSurf predicted the SNP variants F217L, G336D, L321P, and H405P were located at the structural and buried residues, while the remaining highly deleterious AKT1 missense SNPs were at exposed and functional residues.

### 3.5. Predicting the Effects of the Most Deleterious SNPs on the Stability of AKT1 Protein

MUpro predicted that all these SNPs will decrease the stability of the protein. I-Mutant also predicted a similar decrease in stability except in the cases of three mutations—G336D, H265Y, and T312I—where the mutations G336D and H265Y were found to have a neutral effect on the protein’s stability and the mutation Thr312Ile was predicted to increase the protein’s stability. The effect of these SNPs on the AKT1 protein stability as predicted by both I-Mutant and MUpro webservers is listed in Table 2.

### 3.6. Solvent Accessible Area Prediction Using NetSurfP

In our study, the NetSurfP webserver predicted that 10 of the 12 highly deleterious missense SNPs, both the mutated and their respective wild-type variants, were located at buried residues (Table 3). These highly deleterious missense mutations also did not significantly affect the relative surface accessibility for the mutant (MT) and their wild-type (WT) variants.

### 3.7. Predicting the Effects of the Most Deleterious SNPs on the Structure of AKT1 Protein

The Project HOPE server obtained the structural information for predicting the effect of the SNPs on the 3D structure of AKT1 protein from the analysis of PDB ID: 6HHJ. The Project HOPE server predicted that all the mutations are located on the protein kinase domain of the AKT1 protein. It also predicted the effect of the amino acid substitutions on amino acid hydrophobicity, amino acid size, amino acid charge, protein function, protein flexibility, protein interactions, and protein structure. The detailed effects of each highly deleterious AKT1 missense SNPs on the protein structure are given in Table 4.

### 3.8. Protein–Protein Interaction Analysis Using STRING

**Figure 2:**
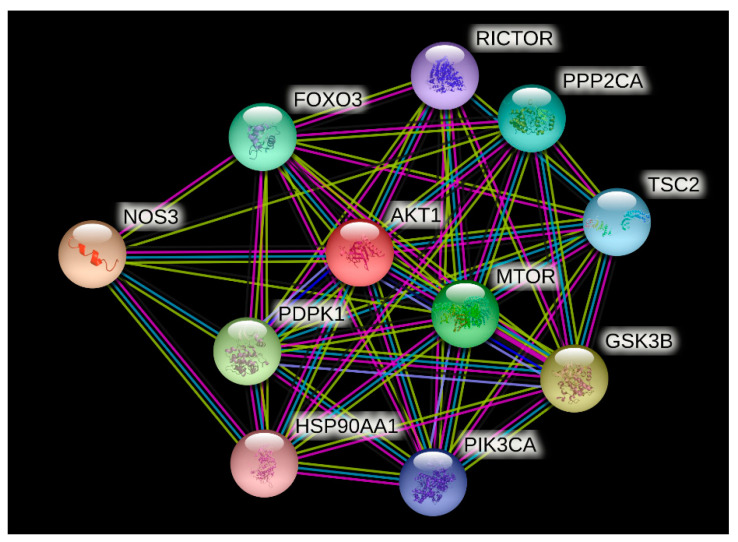
Protein association network of AKT1 protein generated using STRING database (https://string-db.org/, accessed on 31 August 2021).

The STRING analysis showed that the AKT1 protein interacts with nitric oxide synthase (NOS3), glycogen synthase kinase-3 beta (GSK3B), 3-phosphoinositide-dependent protein kinase 1 (PDPK1), serine/threonine-protein kinase mTOR (MTOR), forkhead box protein O3 (FOXO3), serine/threonine-protein phosphatase 2A catalytic subunit alpha isoform (PPP2CA), tuberin (TSC2), phosphatidylinositol 4,5-bisphosphate 3-kinase catalytic subunit alpha isoform (PIK3CA), rapamycin-insensitive companion of mTOR, subunit of mTORC2 (RICTOR), and heat shock protein HSP 90-alpha (HSP90AA1) proteins (refer Figure 2). The STRING server predicted these associations between AKT1 and other proteins on the basis of information available from data mining, different experiments, and databases.

### 3.9. Determining the Highly Deleterious AKT1 Missense SNPs Association with Cancer

The CanSAR Black webserver predicted that the substitution of arginine residue at position 273 with glutamine is moderately associated with liver cancer. For other SNPs, their association with cancer could not be determined.

### 3.10. Analysis of AKT1 Gene Expression in Liver Hepatocellular Carcinoma and Survival Rate

**Figure 3:**
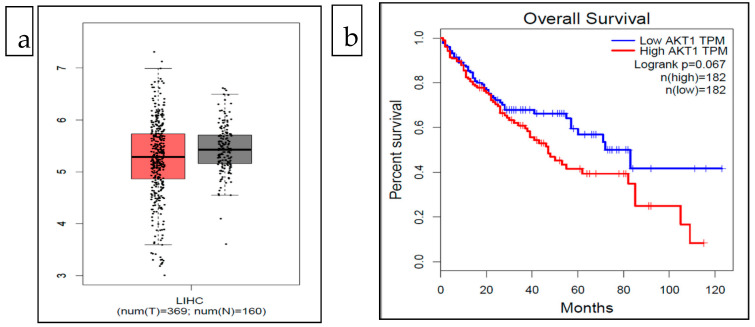
(**a**). Expression analysis of AKT1 gene in cases of LIHC: red (tumor) and gray (normal) (**b**). Overall survival of LIHC patients with low and high expression of AKT1. This figure is generated using GEPIA webserver available at http://gepia.cancer-pku.cn/ accessed on 31 August 2021.

The expression analysis of the AKT1 gene in cases of LIHC (liver hepatocellular carcinoma) using boxplot analysis showed that the case of LIHC had relatively low expression of the AKT gene (Figure 3). The survival analysis showed that the abnormal expression of the AKT1 gene could be related to poor prognosis. The patients in the low expression group had better percent survival in comparison to the high expression group over a period of 120 months (Figure 3). However, a significant association between the expression of the AKT1 gene and the overall survival of LIHC patients could not be established as the logrank *p* value was above 0.05 (Figure 3).

### 3.11. Prediction of 3D Model of the Mutant Proteins

For G157R, 92.72% of residues were in Ramachandran’s favored region and 1.88% of residues were in Ramachandran’s outlier region. For G159V, 91.78% of residues were in Ramachandran’s favored region and 1.64% of residues were in Ramachandran’s outlier region. For G336D, 92.49% of residues were in Ramachandran’s favored region and 1.17% of residues were in Ramachandran’s outlier region. For H265Y, 90.38% of residues were in Ramachandran’s favored region and 1.41% of residues were in Ramachandran’s outlier region.

### 3.12. Wild and Mutant AKT1 Protein’s Structure Comparison

The interactions among the wild-type and four mutant proteins were compared. The results show that there were variations in the number of hydrogen bonds formed by wild-type and mutated residues with other residues in the wild-type and mutant proteins. For the variant G159V, the wild-type residue, glycine, formed two hydrogen bonds with glycine at 162 positions whereas for the mutated protein there was only one hydrogen bond between the mutated residue valine at 159 positions and glycine at 162 positions. In variant G157R, both the wild-type residue (glycine) and mutated residue (arginine) formed two hydrogen bonds each with valine at 164 positions in both wild and mutated proteins. In variant G336D, the wild-type residue glycine at 336 positions formed three hydrogen bonds with tryptophan at 332 positions, glycine at 334 positions, and tyrosine at 340 positions. However, in the mutant protein, one hydrogen bond was lost, and mutated residue aspartic acid at 336 positions formed only two hydrogen bonds with glycine at 334 positions and tyrosine at 340 positions. Similarly, in the variant H265Y wild-type residue, histidine at 265 positions formed five hydrogen bonds with aspartic acid at 262 positions, leucine at 261 positions, asparagine at 269 positions, aspartic acid at 331 positions, and valine at 270 positions. Whereas in the mutant protein, the mutated residue tyrosine formed only two hydrogen bonds with leucine at 261 positions and proline at 51 positions. Furthermore, the structure of wild-type and mutant protein structures were compared by the TM-align tool. The TM-score of the superimposed and mutant AKT1 protein structures suggests that all the structures are in the same fold because for all the superimpositions the TM-score is above 0.5 and below 1. The mutant H265Y showed the highest root mean square deviation suggesting higher structural deviation in comparison to other mutants. The TM-score and RMSD value for the superimposed wild AKT1 protein and mutant AKT1 proteins are shown in Table 5.

### 3.13. Effect of Point Mutations on AKT1 Protein Function

**Figure 4:**
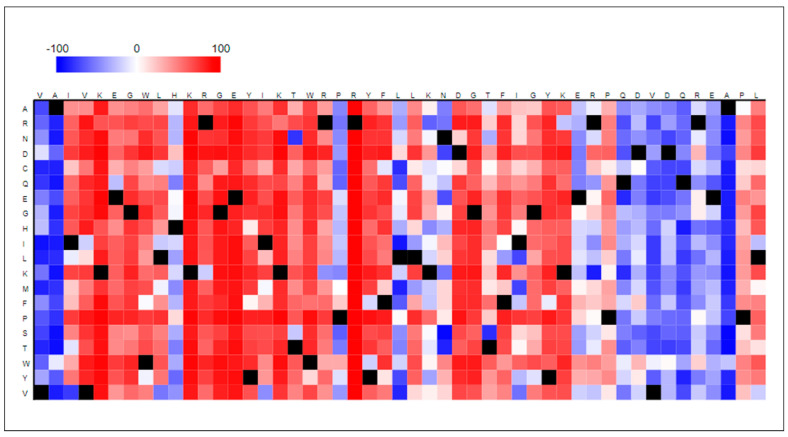
Effect of point mutation on AKT1 protein function represented in the form of a heatmap generated using protr package in R software.

A point mutation simply refers to a single alteration of a residue in a sequence; however, a single change may lead to catastrophes at the genetic level in humans. Therefore, it becomes mandatory to check and identify the potential of a macromolecule to have point mutations in its sequences. Functional effects of single amino acid residue substitutions/alterations have been identified and represented in the form of a heatmap (refer Figure 4). The red-colored blocks represent a greater value (>50) that refers to a higher possibility of showing mutation effects, whereas white blocks (value −50 < score < 50) represent a poor or minimal possibility of showing mutational effects. Blue-colored blocks, on the other hand, indicate a low score (score < −50) showing no residual effect, while black-colored blocks represent wild-type amino acid residues.

It is evident that AKT1 is susceptible to showcasing strong altered effects on its functioning in the case of a single amino acid alteration in its sequence. Every residue in the AKT1 protein sequence corresponds to showcase an effect with a greater score (>50). This result validates our point that the greater the number of mutations in AKT1, the poorer is the stability of the structure. Supplementary Table S1 is provided that encapsulates the residue-wise score and effect of the AKT1 protein sequence.

### 3.14. Prediction of AKT1 Protein–Ligand Binding Sites

The 3DligandSite webserver predicted the residues at positions 161, 177, 179, 211, 227, 228, 229, 230, 278, 279, 281, 291, and 292 as ligand binding sites on the AKT1 protein. Similarly, the COACH webserver predicted the residues at positions 156, 157, 158, 164, 177, 179, 211, 227, 228, 229, 230, 234, 278, 279, 281, 291, 292, and 438 as ligand binding sites on the AKT1 protein. The residues at positions 177, 179, 211, 227, 228, 229, 230, 278, 279, 281, 291, and 292 were predicted as ligand binding sites by both webservers. The COACH webserver predicted the residue at 157 is involved in ligand binding. Hence, the SNP G157R could affect the ligand binding in the mutant AKT1 protein. To further assess the impact of the mutations on ligand binding, a molecular docking study was performed.

### 3.15. Molecular Docking Analysis

The molecular docking study showed that the interactions between ligand and receptor proteins were not affected significantly by the mutations (Table 6). In the WT protein–ligand complex, the global energy was −90.57 kcal/mol. Global energy is the binding energy of the complex. The global energy among the mutant protein–ligand complexes was almost similar to the WT-ligand docking, except for the G336D mutant protein–ligand complex.

### 3.16. Analysis of the Effect of 3′UTR SNPs on miRNA Binding Sites

Using PolymiRTS, the association between miRNAs and 14 3′UTR SNPs was determined. Among these variants, two SNPs were identified as disrupting the conserved site of miRNAs. Additionally, for four SNPs the ancestral allele could not be determined. Interestingly, three SNPs were found to be involved in the creation of a new miRNA site. Finally, five SNPs were determined to either disrupt the conserved site of miRNAs or create new miRNA sites. The list of 3′UTR SNPs of the AKT1 gene that affect miRNA is provided in Supplementary Table S3.

### 3.17. Analysis of Association of AKT1 SNPs with Different Cancers

**Figure 5:**
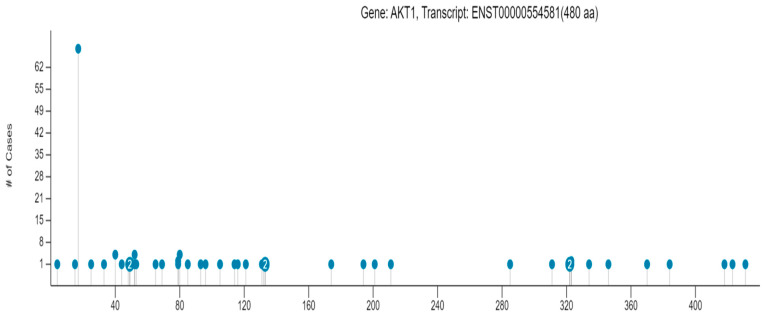
Position of missense SNPs on the AKT1 protein and the number of cancer cases affected by the SNPs. This figure is generated using GDC portal (https://portal.gdc.cancer.gov/ accessed on 9 June 2023).

In the GDC portal, information about 99 mutations in the AKT1 protein is available, out of which 50 mutations are missense SNPs. The position of different missense SNPs on the AKT1 protein and the number of cancer cases associated with the individual AKT1 SNPs are shown in Figure 5. These 50 SNPs affected 126 cases. Altogether, 55 cases of adenomas and adenocarcinomas, 32 cases of ductal and lobular neoplasms, 15 cases of squamous cell neoplasms, 5 cases of nevi and melanomas, 5 cases of cystic, mucinous, and serous neoplasms, 4 cases of lymphoid leukemia, 4 cases of transitional cell papilloma, 3 cases of glioma, 2 cases of complex epithelial neoplasms, and 1 case of thymic epithelial neoplasm were affected by the AKT1 SNPs. In Supplementary Table S4, different AKT1 missense SNPs, the number of cases in the cohort, the number of cases across GDC, and the number of different cancer types affected by the AKT1 SNPs are provided.

## 4. Discussion

With the advancement in high-throughput sequencing technologies, the identification of genomic variants in the human genome has increased rapidly. The dbSNP database maintained by NCBI (National Center for Biotechnology Information) contains more than 1 billion SNPs and 9.6 million of these SNPs are missense. The missense SNPs can influence disease susceptibility, alter protein structure and function, and are relevant in various pathological conditions [57]. The determination of these SNPs, if they are deleterious or harmless, to the protein function and structure is important. Furthermore, the analysis of the SNPs and their associations with human diseases are also imperative. However, the characterization of these SNPs as harmful or neutral and determining their associations with the disease can be an arduous, time-consuming, and costly job [27]. The in silico analysis of the SNPs can be useful in overcoming these limitations and filtering out the SNPs which can be most deleterious to the protein function and structure and have an association with diseases. Previously, the in silico approach has been used to assess the impact of the SNPs on various proteins [58,59,60].

AKT1 is a serine/threonine kinase belonging to the protein kinase B family [61]. AKT1 is necessary for the mediation of apoptosis, angiogenesis, metabolism, and cell proliferation in normal as well as cancerous cells. The AKT1 gene in an oncogene and mutations in it can cause cancer. The mutations in the AKT1 gene have been associated with different types of cancer, such as lung, colorectal, ovarian, and breast cancer [62,63]. Furthermore, the AKT1 gene mutations are also associated with Proteus syndrome, Cowden, and Cowden-like syndromes [64,65]. Hence, it is imperative to study the mutations in the AKT1 gene. In this study, the deleterious missense SNPs of the AKT1 gene were identified and their effect on its function and structure was also assessed using various computational tools. Furthermore, the association of these highly deleterious missense SNPs with different forms of cancers was also analyzed using an in silico approach. Previously, different studies have shown the association between SNP E17K breast cancer, gastric cancer, and squamous cell lung cancer [66,67,68]. Two different in silico studies have been performed previously to identify deleterious missense SNPs in the AKT1 gene. Shanthi et al. predicted, using an in silico approach, that the missense SNPs E17K, E17S, E319G, L357P, and P388T can affect protein function [69]. Khan and Ansari also predicted the SNPs E17K, P388T, E319G, A399T, D32E, and A255T as deleterious [16]. However, these two studies analyzed very few numbers of SNPs. It also analyzed only 22 missense SNPs, whereas Khan and Ansari studied 29 nsSNPs. Years have passed since the previous in silico studies on SNPs of AKT1, and with time the number of missense SNPs of the AKT1 gene has also increased. This study has analyzed 10 times the number of missense AKT1 SNPs than in comparative studies [16]. Furthermore, the number of computational tools to identify the deleterious missense AKT1 SNPs is more (n = 8) in comparison to the two previous studies (n = 4). The algorithms used by the eight webservers to predict the deleterious SNPs are mostly different from each other. Hence, the SNPs which are predicted to be deleterious by all the servers are more likely to be reliable [27].

The data for 293 missense AKT1 gene SNPs were obtained from the dbSNP database. Then, these SNPs were filtered out to predict the highly deleterious SNPs using eight different tools, namely, PROVEAN, SIFT, POLYPHEN, SNAP2, Mutation Assessor, PONP-2, PANTHER, and PMUT. The combination of these different webservers will improve the accuracy and reliability of the prediction. Overall, 12 SNPs—G157R, H405P, G159V, F217L, G336D, R391G, H265Y, R273Q, L321P, G345S, T312I, and G286R—were predicted as highly deleterious from the set of 293 AKT1 missense SNPs. Hence, these 12 SNPs were selected for further analysis. Next, ConSurf analysis was performed to predict the evolutionarily conserved residues in the protein as the conserved residues can be important for protein folding, structural stability, and function [69]. The ConSurf analysis predicted that 11 out of the 12 SNPs were located at highly conserved residues as they have the highest ConSurf score of 9. Furthermore, the ConSurf analysis also predicted that all these 12 SNPs were located at either structurally or functionally important residues. This analysis further corroborates that these 12 SNPs are likely to be highly deleterious and affect the function and structure of the protein.

To further validate the in silico results, the effect of these 12 highly deleterious SNPs on the stability of the AKT1 protein was predicted. Maintaining protein stability is crucial for the proper structure and functioning of a protein [70]. The stability of a protein is responsible for its conformational arrangement, which ultimately dictates the protein’s function. Any changes in protein stability can lead to protein misfolding, degradation, or abnormal aggregation [71]. Two different tools, I-Mutant and MUpro, were used to predict the effect of these highly deleterious SNPs on the stability of the AKT1 protein to improve the reliability of the results. These two webservers unanimously predicted that 8 out of the 12 highly deleterious SNPs will decrease the stability of the AKT1 protein. However, for the remaining four SNPs, the results varied between the MUpro and I-Mutant server. MUpro predicted the remaining four SNPs to decrease the stability, whereas I-Mutant predicted that two of the SNPs will have a neutral effect and two others will increase the stability of the AKT1 protein.

The solvent-accessible area refers to an area of a protein that can come in contact with polar solvents such as water. The analysis of the surface accessible area of amino acids could be of significance during the functional annotation of disease-associated SNP variant proteins [72]. The SNPs occurring at buried residues are more frequently associated with diseases in comparison to SNPs occurring at exposed residues [72]. Hence, the solvent-accessible area was predicted using NetSurfP analysis which showed that 10 of the 12 highly deleterious AKT1 missense SNPs are located at buried residues. The effect of these SNPs on the structure of the AKT1 protein was predicted by the Project HOPE webserver. The Project HOPE server predicted that all 12 deleterious SNPs are located at the protein kinase domain of AKT1 protein. All 12 highly deleterious SNPs were predicted to affect the structure of the AKT1 protein. They were also predicted to affect the interactions and flexibility of the protein. The HOPE webserver also predicted four SNPs, G157R, G159V, G336D, and H265Y, are located at 100% conserved residues. As these four residues were also predicted as highly conserved by the ConSurf webserver (ConSurf score = 9) and mutations at conserved residues can impact the function and structure of proteins, these four SNPs were selected for further analysis. Further molecular docking analysis of these four mutant and WT proteins with AKT inhibitor VIII was performed to determine the effect of SNPs on the binding of the ligand and receptor molecules. The molecular docking study showed similar global energies for all the ligand and protein complexes implying that the binding of the ligand and receptor is not affected significantly. It was predicted that one of the mutations, G157R, could affect the ligand binding to the AKT1 protein as the residue at 157 positions was predicted to be involved in ligand binding using the COACH webserver. However, further in vitro studies are needed to corroborate the effect of SNPs on the ligand-binding ability of the AKT1 protein. Previously, an in vitro study has reported the increased affinity of E17K AKT1 mutant protein to PI(4,5)P_2_, a plasma membrane protein [73]. Similarly, Wu et al. reported in another in vitro study that the AKT1 inhibitor VIII is five times less effective on the E17K AKT1 mutant protein in comparison to the WT AKT1 protein [50].

After identifying the highly deleterious AKT1 missense SNPs and predicting their effect on the structure and function of AKT1 protein, their association with human diseases was predicted. The STRING webserver showed that the AKT1 protein interacts with different proteins belonging to diverse biological pathways. Hence, the mutations in the AKT1 protein are likely to affect interactions of the AKT1 protein with other important proteins and affect different pathways, thus resulting in different diseases. By analyzing the effect of the point mutation in AKT1 functioning, we discerned and validated that AKT1 is susceptible to showcasing strong altered effects on its functioning in the case of a single amino acid alteration in its sequence. Therefore, the greater the mutations in AKT1, the poorer is the stability with major disordered regions in its structure, thus causing insignificant and altered functioning. This may lead to the progression of several genetic diseases in humans.

Different studies have shown the association of mutations in the AKT1 gene with Proteus syndrome, Cowden syndrome, and cancer [63,64,65]. As the mutations in AKT1 protein have been associated with different types of cancer, the association of highly deleterious AKT1 missense SNPs predicted in this study with cancer was further explored. The CanSAR Black webserver predicted that only one of the highly deleterious SNPs (R273Q) was moderately associated with liver cancer. However, there is a possibility that other deleterious missense SNPs predicted in this study might be associated with cancer and other diseases. Hence, further intensive in vivo experiments are necessary to determine the association of the other highly deleterious AKT1 missense SNPs with other human diseases. As the SNP was associated with liver cancer, the role of change in the expression of the AKT1 gene in LIHC was also explored. The results in our study showed that the abnormal expression of the AKT1 gene might affect the survival of the patients. Previous studies have shown that the differential regulation of AKT1 contributes to the development, progression, proliferation, and survival of hepatocellular carcinoma [74,75].

miRNAs, which are short non-coding RNAs approximately 22 nucleotides in length, play a crucial role as post-transcriptional regulators. They achieve this by binding to the 3′UTRs of messenger RNAs (mRNAs) [76]. The effectiveness of miRNA targeting largely relies on the binding between miRNA and its complementary target site. However, sequence variations, such as SNPs, in either miRNAs or their target sites can disrupt this binding process [53]. Consequently, these variations can have significant consequences on miRNA function, leading to notable effects on gene expression and higher-level phenotypes [53]. These miRNA-related polymorphisms have been linked to various human diseases, including diabetes [77], cancers [78], Alzheimer’s disease [79], and Parkinson’s disease [80]. This study predicted 14 different 3′UTR in the AKT1 gene which could create or disrupt miRNA sites and affect miRNA–mRNA interactions [76].

## 5. Conclusions

After the extensive analysis of 293 AKT1 missense SNPs using a multitude of in silico tools, 12 highly deleterious SNPs were identified. These missense SNPs can affect the structure and functions of the AKT1 protein. Out of the 12 highly deleterious SNPs, 4 SNPs, G157R, G159V, G336D, and H265Y, were predicted to be located at highly conserved residues. It was also predicted that one of the highly deleterious SNPs, G157R, could affect the ligand binding to AKT1 protein as the residue at 157 positions was predicted to be involved in ligand binding using the COACH webserver. Another highly deleterious SNP, R273Q, was predicted to be associated with liver cancer. The deleterious effect of these SNPs is being reported for the first time in this study. As these results suggest, the potential of the deleterious missense SNPs to affect the structure and function of AKT1, are based on in silico experiments. Further in vitro, in vivo, and population-based studies are required to corroborate these findings. This study can be useful for pharmacogenomics, molecular diagnosis of diseases, and developing inhibitors of the AKT1 oncogene.

## Figures and Tables

**Table 1:** List of deleterious nsSNPs that were predicted to have a functional effect on AKT1 protein by all the webservers used in this study. (PRO = PROVEAN Score, POLY = POLYPHEN Score, MA = Mutation Assessor, PD = Probably damaging).

dbSNP ID	Substitution	PRO	SIFT Score	POLY Score	SNAP 2 Score	MA Score	PANTHER	PON-P2 Score	PMUT Score
rs568870136	G157R	−7.48	0.001	1.000	91	4.01	PD	0.852	0.90
rs768660759	H405P	−9.44	0.001	0.790	86	3.96	PD	0.878	0.90
rs866169013	G159V	−8.37	0.000	0.967	90	4.815	PD	0.860	0.90
rs971568277	F217L	−5.29	0.007	0.916	39	1.935	PD	0.868	0.89
rs1197941200	G336D	−6.84	0.000	1.000	89	4.64	PD	0.895	0.90
rs1394191738	R391G	−6.53	0.001	1.000	87	4.475	PD	0.834	0.90
rs1397852501	H265Y	−5.61	0.000	0.999	13	3.61	PD	0.826	0.90
rs1424916218	R273Q	−3.45	0.032	0.999	81	3.635	PD	0.87	0.90
rs1454909926	L321P	−6.61	0.000	1.000	49	3.72	PD	0.917	0.90
rs1892483378	G345S	−5.48	0.033	0.992	30	2.91	PD	0.766	0.81
rs1892502921	T312I	−5.53	0.000	0.984	82	3.595	PD	0.833	0.90
rs1892506436	G286R	−7.21	0.032	0.999	82	2.27	PD	0.856	0.90

**Table 2:** Prediction of AKT1 missense SNPs on the stability of AKT1 protein.

Mutation	I-Mutant Prediction	DDG Value (kcal/mol)	MUpro Stability Prediction	ΔG Value
G157R	Large decrease in stability	−0.47	Decrease stability	−0.74
H405P	Large increase in stability	0.40	Decrease stability	−1.08
G159V	Large decrease in stability	−0.24	Decrease stability	−0.43
F217L	Large decrease in stability	−1.37	Decrease stability	−0.92
G336D	Neutral effect	−0.66	Decrease stability	−0.39
R391G	Large decrease in stability	−0.93	Decrease stability	−1.24
H265Y	Neutral	−0.01	Decrease stability	−0.19
R273Q	Large decrease in stability	−0.90	Decrease stability	−1.23
L321P	Large decrease in stability	−1.92	Decrease stability	−1.38
G345S	Large decrease in stability	−1.16	Decrease stability	−0.77
T312I	Large increase in stability	0.08	Decrease stability	−0.31
G286R	Large decrease in stability	−0.44	Decrease stability	−0.63

**Table 3:** Prediction of surface accessibility of the mutant and wild-type variants.

Substitution	Surface Accessibility	Relative Surface Accessibility (WT) in %	Relative Surface Accessibility (MT) in %
G157R	Exposed for both MT and WT	27	30
H405P	Buried for both MT and WT	9	10
G159V	Exposed for both MT and WT	33	38
F217L	Buried for both MT and WT	5	8
G336D	Buried for both MT and WT	3	4
R391G	Buried for both MT and WT	13	18
H265Y	Buried for both MT and WT	4	4
R273Q	Buried for both MT and WT	13	14
L321P	Buried for both MT and WT	5	6
G345S	Buried for both MT and WT	10	17
T312I	Buried for both MT and WT	13	12
G286R	Buried for both MT and WT	3	6

**Table 4:** Project HOPE server analysis of the effects of most deleterious missense AKT1 SNPs on the 3D structure of AKT1 protein.

Substitution	Effects of the Mutation
G157R	The mutation can affect the flexibility of the protein, cause loss of interactions with the nucleotide, affect the function of the protein, and disturb the local structure. The mutation is at a 100% conserved residue which can be damaging to the protein. The mutation also introduces a charge in a buried residue which can lead to protein-folding problems.
H405P	The mutation will cause a loss of external interactions due to the inability of mutant residue to form hydrogen bonds as the wild-type and mutant residues differ in size and hydrophobicity. The mutation can also disturb domain and protein function.
G159V	The wild-type residue was buried in the core of the protein. The mutant residue is bigger and probably will not fit. The torsion angles for this residue are unusual. Only glycine is flexible enough to make these torsion angles; mutation into another residue will force the local backbone into an incorrect conformation and will disturb the local structure. The differences between the wild-type and mutant residue can disturb the core structure of this important domain and thereby affect the catalytic activity. The mutation is at a 100% conserved residue which can be damaging to the protein.
F217L	The wild-type and mutant amino acids differ in size and can cause empty space in the core of the protein. The mutation might affect the function of the protein.
G336D	The wild-type and mutant residues differ in size and charge. The mutant residue introduces a charge in a buried residue which can lead to protein-folding problems. The wild-type residue was buried in the core of the protein. The mutant residue is bigger and probably will not fit. The mutation can also cause a loss of flexibility as the wild-type residue is glycine, which is the most flexible of all residues. The mutation is at a 100% conserved residue which can be damaging to the protein.
R391G	The wild-type and mutant residues differ in charge, size, and hydrophobicity. The charge of the buried wild-type residue is lost by this mutation. The mutation will cause an empty space in the core of the protein and also cause a loss of hydrophobic interactions in the core of the protein. The mutation can also cause loss of function of the protein. The mutation introduces glycine, which can affect the rigidity of the protein at this position.
H265Y	The mutation is at a 100% conserved residue which can be damaging to the protein. The wild-type and mutant residues differ in size and hydrophobicity. The wild-type residue was buried in the core of the protein. The mutant residue is bigger and probably will not fit. The mutation can cause a loss of hydrogen bonds in the core of the protein and disturb its correct folding. The mutation can also affect the function of the protein.
R273Q	The wild-type and mutant residues differ in size and hydrophobicity. As the mutant residue is smaller than the wild-type residue it will cause an empty space in the core of the protein. The charge of the buried wild-type residue is lost by this mutation. The mutation can also affect the function of the protein by changing the local structure surrounding the active site as the mutated residue is located very close to the active site.
L321P	The wild-type and mutant residues differ in size. As the mutant residue is smaller than the wild-type residue, it will cause an empty space in the core of the protein. The mutation can also affect the function of the protein.
G345S	The wild-type and mutant residues differ in size. The residue is located on the surface of the protein, and mutation of this residue can disturb interactions with other molecules or other parts of the protein. The mutation introduces an amino acid with different properties, which can disturb this domain and abolish its function.
T312I	The mutant residue is bigger than the wild-type residue. The mutant residue is more hydrophobic than the wild-type residue. The wild-type residue forms a hydrogen bond with glutamic acid at position 314. The size difference between the wild-type and mutant residue means that the new residue is not in the correct position to make the same hydrogen bond as the original wild-type residue did. The mutation can affect the function of the protein.
G286R	There is a difference in charge between the wild-type and mutant amino acid. The mutant residue introduces a charge in a buried residue which can lead to protein-folding problems. The wild-type and mutant amino acids differ in size. The mutant residue is bigger than the wild-type residue. The wild-type residue was buried in the core of the protein. The mutant residue is bigger and probably will not fit. The mutation can affect the function of the protein.

**Table 5:** Structure comparison of wild-type and mutant proteins by TM-align.

Superimposition	TM-Score	RMSD Value
Wild and G157R mutant protein	0.99925	0.20
Wild and G159V mutant protein	0.99894	0.23
Wild and G336D mutant protein	0.99880	0.28
Wild and H265Y mutant protein	0.99841	0.31

**Table 6:** Molecular docking analysis.

Docked Complex	Global Energy (kcal/mol)
WT protein and AKT inhibitor VIII	−90.57
G157R mutant protein and AKT inhibitor VIII	−89.01
G159V mutant protein and AKT inhibitor VIII	−90.10
G336D mutant protein and AKT inhibitor VIII	−105.00
H265Y mutant protein and AKT inhibitor VIII	−91.18

## Data Availability

Available on request.

## Supplementary Materials

The following supporting information can be downloaded at: https://www.mdpi.com/article/10.3390/life13071532/s1, Table S1: List of missense SNPs in the AKT1 gene; Table S2: Prediction of the functional effect of SNPs by different servers. (B = Benign; DG = Damaging; DL = Deleterious; D = Disease; E = Effect; H = High; L = Low; M = Medium; MA = Mutation assessor; N = Neutral, P = Pathogenic; PSD: Possibly Damaging; PB = Probably benign; PD = Probably damaging; S = score; TL = Tolerated and U = Unknown); Table S3: Analysis of the effect of 3′ UTR SNPs on microRNA (miRNA) binding sites using PolymiRT analysis for gene AKT1. The functional classes “C” means the derived allele creates a new miRNA site, “D” means the derived allele disrupts a con-served miRNA site, “N” means the derived allele disrupts a nonconserved miRNA site, and “O” means it is not possible to determine the ancestral allele; Table S4: Information of AKT1 missense SNPs available from the Genomic Data Commons Data Portal (https://portal.gdc.cancer.gov/ accessed on 9 June 2023).

## Abbreviations

GPCR	G-protein coupled receptors
GEPIA	Gene expression profiling interactive analysis
ILK	Integrin-linked kinase
LIHC	Liver hepatocellular carcinoma
MT	Mutant-type variants
NCBI	National center for biotechnology information
PDB	Protein databank
PTM	Post-translational modifications
RTK	Receptor tyrosine kinases
RMSD	Root mean square deviation
SNP	Single nucleotide polymorphism
SVM	Support vector machine
UTR	Untranslated region
WT	Wild-type variants
