# Supplementary material for: Discovering Deleterious Single Nucleotide Polymorphisms of Human AKT1 Oncogene: An In Silico Study
Source: Life (Basel). 2023 Jul 10;13(7):1532. doi: 10.3390/life13071532 (PMC10381612; doi:10.3390/life13071532)
Supplement: Supplementary file 1 [file life-13-01532-s001.zip › Supplementary Table S1.pdf]

Supplementary Table S1: List of missense SNPs in the AKT1 gene

S.No.	dbSNP ID	Location	Change
1	rs113547523	14:104770390 (GRCh38)<br>14:105236727 (GRCh37)	C to T
2	rs121434592	14:104780214 (GRCh38)<br>14:105246551 (GRCh37)	C to T
3	rs140532443	14:104792637 (GRCh38)<br>14:105258974 (GRCh37)	C to T
4	rs144112075	14:104770395 (GRCh38)<br>14:105236732 (GRCh37)	G to A<br>Or G to T
5	rs146483593	14:104772397 (GRCh38)<br>14:105238734 (GRCh37)	C to T
6	rs146875699	14:104780125 (GRCh38)<br>14:105246462 (GRCh37)	G to T
7	rs183989506	14:104776740 (GRCh38)<br>14:105243077 (GRCh37)	C to T
8	rs188580689	14:104772430 (GRCh38)<br>14:105238767 (GRCh37)	C to T
9	rs374093099	14:104780121 (GRCh38)<br>14:105246458 (GRCh37)	G to A
10	rs375395037	14:104773520 (GRCh38)<br>14:105239857 (GRCh37)	C to T
11	rs375990114	14:104775657 (GRCh38)<br>14:105241994 (GRCh37)	G to A<br>G to C
12	rs397514644	14:104780190 (GRCh38)<br>14:105246527 (GRCh37)	G to A
13	rs397514645	14:104770805 (GRCh38)<br>14:105237142 (GRCh37)	T to G
14	rs549083521	14:104773486 (GRCh38)<br>14:105239823 (GRCh37)	G to A
15	rs587778018	14:104770411 (GRCh38)<br>14:105236748 (GRCh37)	A to G or A to T
16	rs587778019	14:104776721 (GRCh38)<br>14:105243058 (GRCh37)	G to C or G to T
17	rs745803788	14:104770394 (GRCh38)<br>14:105236731 (GRCh37)	C to T
18	rs746272761	14:104772941 (GRCh38)<br>14:105239278 (GRCh37)	C to G or C to T
19	rs754031503	14:104792634 (GRCh38)<br>14:105258971 (GRCh37)	C to A or C to T
20	rs758157217	14:104780156 (GRCh38)<br>14:105246493 (GRCh37)	A to G
21	rs758456890	14:104775203 (GRCh38)<br>14:105241540 (GRCh37)	A to G
22	rs759702315	14:104770747 (GRCh38)<br>14:105237084 (GRCh37)	T to C
23	rs762705090	14:104772951 (GRCh38)<br>14:105239288 (GRCh37)	G to A or T
24	rs764863282	14:104772373 (GRCh38)<br>14:105238710 (GRCh37)	C to T
25	rs768070795	14:104775131 (GRCh38)<br>14:105241468 (GRCh37)	G to A
26	rs768606668	14:104773562 (GRCh38)<br>14:105239899 (GRCh37)	G to A
27	rs771065764	14:104776719 (GRCh38)<br>14:105243056 (GRCh37)	C to T

28	rs773607483	14:104772427 (GRCh38)<br>14:105238764 (GRCh37)	T to C
29	rs774836044	14:104780120 (GRCh38)<br>14:105246457 (GRCh37)	C to T
30	rs778376616	14:104775681 (GRCh38)<br>14:105242018 (GRCh37)	C to T
31	rs780207480	14:104775001 (GRCh38)<br>14:105241338 (GRCh37)	G to A or C
32	rs780571834	14:104773532 (GRCh38)<br>14:105239869 (GRCh37)	G to A
33	rs781339141	14:104780157 (GRCh38)<br>14:105246494 (GRCh37)	T to C
34	rs781388586	14:104772938 (GRCh38)<br>14:105239275 (GRCh37)	G to A or T
35	rs983007851	14:104775123 (GRCh38)<br>14:105241460 (GRCh37)	G to A
36	rs1057518602	14:104776710 (GRCh38)<br>14:105243047 (GRCh37)	T to C
37	rs1057519804	14:104776711 (GRCh38)<br>14:105243048 (GRCh37)	G to T
38	rs1060503071	14:104775656 (GRCh38)<br>14:105241993 (GRCh37)	C to T
39	rs1159942120	14:104770788 (GRCh38)<br>14:105237125 (GRCh37)	C to A
40	rs1205616929	14:104770352 (GRCh38)<br>14:105236689 (GRCh37)	C to T
41	rs1334042967	14:104772434 (GRCh38)<br>14:105238771 (GRCh37)	C to A or T
42	rs1360111387	14:104773301 (GRCh38)<br>14:105239638 (GRCh37)	C to T
43	rs1457484217	14:104775725 (GRCh38)<br>14:105242062 (GRCh37)	C to T
44	rs1458420660	14:104773538 (GRCh38)<br>14:105239875 (GRCh37)	G to A or T
45	rs1555383354	14:104772383 (GRCh38)<br>14:105238720 (GRCh37)	C to G
46	rs1555383471	14:104773291 (GRCh38)<br>14:105239628 (GRCh37)	A to G
47	rs1555383511	14:104773513 (GRCh38)<br>14:105239850 (GRCh37)	A to G
48	rs1555383695	14:104775098 (GRCh38)<br>14:105241435 (GRCh37)	T to C
49	rs1566815164	14:104770847 (GRCh38)<br>14:105237184 (GRCh37)	G to A
50	rs1566816289	14:104772891 (GRCh38)<br>14:105239228 (GRCh37)	C to T
51	rs1566818099	14:104775731 (GRCh38)<br>14:105242068 (GRCh37)	T to C
52	rs1566826869	14:104792640 (GRCh38)<br>14:105258977 (GRCh37)	T to C
53	rs1595243377	14:104773922 (GRCh38)<br>14:105240259 (GRCh37)	T to C
54	rs11555431	14:104772888 (GRCh38)<br>14:105239225 (GRCh37)	G to T
55	rs11555432	14:104772980 (GRCh38)<br>14:105239317 (GRCh37)	A to G
56	rs11555433	14:104775143 (GRCh38)<br>14:105241480 (GRCh37)	A to G
57	rs11555435	14:104770403 (GRCh38)	C to A

		14:105236740 (GRCh37)	
58	rs12881616	14:104773252 (GRCh38)<br>14:105239589 (GRCh37)	T to C
59	rs142843688	14:104775660 (GRCh38)<br>14:105241997 (GRCh37)	G to A
60	rs143266084	14:104772408 (GRCh38)<br>14:105238745 (GRCh37)	C to T
61	rs144128670	14:104775081 (GRCh38)<br>14:105241418 (GRCh37)	C to T
62	rs201291259	14:104770348 (GRCh38)<br>14:105236685 (GRCh37)	G to A
63	rs201636005	14:104780167 (GRCh38)<br>14:105246504 (GRCh37)	A to C or G
64	rs368797346	14:104792600 (GRCh38)<br>14:105258937 (GRCh37)	C to T
65	rs369198922	14:104775726 (GRCh38)<br>14:105242063 (GRCh37)	G to A or T
66	rs369520527	14:104772950 (GRCh38)<br>14:105239287 (GRCh37)	C to T
67	rs369698909	14:104773949 (GRCh38)<br>14:105240286 (GRCh37)	C to T
68	rs371467719	14:104773497 (GRCh38)<br>14:105239834 (GRCh37)	G to C
69	rs372502847	14:104772963 (GRCh38)<br>14:105239300 (GRCh37)	T to C
70	rs373253729	14:104772927 (GRCh38)<br>14:105239264 (GRCh37)	C to T
71	rs531850885	14:104772436 (GRCh38)<br>14:105238773 (GRCh37)	C to T
72	rs532268608	14:104780122 (GRCh38)<br>14:105246459 (GRCh37)	T to A or G or C
73	rs549370342	14:104772942 (GRCh38)<br>14:105239279 (GRCh37)	G to A or T
74	rs551254461	14:104773950 (GRCh38)<br>14:105240287 (GRCh37)	G to A
75	rs568870136	14:104775174 (GRCh38)<br>14:105241511 (GRCh37)	C to G
76	rs745500951	14:104775089 (GRCh38)<br>14:105241426 (GRCh37)	A to C or T
77	rs745809388	14:104773537 (GRCh38)<br>14:105239874 (GRCh37)	C to T
78	rs746397537	14:104773079 (GRCh38)<br>14:105239416 (GRCh37)	T to C
79	rs746934495	14:104775722 (GRCh38)<br>14:105242059 (GRCh37)	G to A
80	rs748789094	14:104775128 (GRCh38)<br>14:105241465 (GRCh37)	G to A
81	rs749186394	14:104770819 (GRCh38)<br>14:105237156 (GRCh37)	G to A
82	rs749544983	14:104772409 (GRCh38)<br>14:105238746 (GRCh37)	G to A
83	rs750653493	14:104780162 (GRCh38)<br>14:105246499 (GRCh37)	G to T
84	rs751232562	14:104773525 (GRCh38)<br>14:105239862 (GRCh37)	T to C
85	rs751243134	14:104776752 (GRCh38)<br>14:105243089 (GRCh37)	G to A
86	rs751416672	14:104774984 (GRCh38)<br>14:105241321 (GRCh37)	A to T

87	rs751976958	14:104770770 (GRCh38)<br>14:105237107 (GRCh37)	C to A
88	rs753190812	14:104770753 (GRCh38)<br>14:105237090 (GRCh37)	G to T
89	rs753360468	14:104772367 (GRCh38)<br>14:105238704 (GRCh37)	T to C
90	rs753765116	14:104780142 (GRCh38)<br>14:105246479 (GRCh37)	G to A
91	rs754040453	14:104770420 (GRCh38)<br>14:105236757 (GRCh37)	T to C
92	rs754500025	14:104772369 (GRCh38)<br>14:105238706 (GRCh37)	T to C
93	rs755597789	14:104770816 (GRCh38)<br>14:105237153 (GRCh37)	G to A
94	rs756293014	14:104774993 (GRCh38)<br>14:105241330 (GRCh37)	G to A
95	rs756697784	14:104773531 (GRCh38)<br>14:105239868 (GRCh37)	C to T
96	rs757963528	14:104770363 (GRCh38)<br>14:105236700 (GRCh37)	T to G
97	rs758025607	14:104773502 (GRCh38)<br>14:105239839 (GRCh37)	G to C
98	rs758476416	14:104772380 (GRCh38)<br>14:105238717 (GRCh37)	G to A or C
99	rs759031755	14:104775713 (GRCh38)<br>14:105242050 (GRCh37)	G to A
100	rs759902535	14:104770413 (GRCh38)<br>14:105236750 (GRCh37)	G to C
101	rs760044481	14:104776732 (GRCh38)<br>14:105243069 (GRCh37)	T to C or G
102	rs760384228	14:104773970 (GRCh38)<br>14:105240307 (GRCh37)	T to C
103	rs760536822	14:104776660 (GRCh38)<br>14:105242997 (GRCh37)	G to A
104	rs761151169	14:104775786 (GRCh38)<br>14:105242123 (GRCh37)	T to C
105	rs762040581	14:104773953 (GRCh38)<br>14:105240290 (GRCh37)	C to G or T
106	rs762559261	14:104776747 (GRCh38)<br>14:105243084 (GRCh37)	G to A
107	rs764514218	14:104770769 (GRCh38)<br>14:105237106 (GRCh37)	T to C
108	rs764931115	14:104780148 (GRCh38)<br>14:105246485 (GRCh37)	T to C
109	rs765264778	14:104770749 (GRCh38)<br>14:105237086 (GRCh37)	G to C
110	rs766000895	14:104780141 (GRCh38)<br>14:105246478 (GRCh37)	C to T
111	rs766546254	14:104792631 (GRCh38)<br>14:105258968 (GRCh37)	C to T
112	rs766798551	14:104772435 (GRCh38)<br>14:105238772 (GRCh37)	T to G
113	rs767699129	14:104773346 (GRCh38)<br>14:105239683 (GRCh37)	T to C
114	rs768660759	14:104772411 (GRCh38)<br>14:105238748 (GRCh37)	T to G
115	rs768800433	14:104775762 (GRCh38)<br>14:105242099 (GRCh37)	C to A or G or T
116	rs768898540	14:104780114 (GRCh38)	G to A

		14:105246451 (GRCh37)	
117	rs769619023	14:104775110 (GRCh38)<br>14:105241447 (GRCh37)	A to G
118	rs770370100	14:104770391 (GRCh38)<br>14:105236728 (GRCh37)	G to A or C
119	rs770980034	14:104773580 (GRCh38)<br>14:105239917 (GRCh37)	G to A or C
120	rs772473246	14:104776690 (GRCh38)<br>14:105243027 (GRCh37)	G to A
121	rs773520823	14:104773564 (GRCh38)<br>14:105239901 (GRCh37)	G to C
122	rs773971502	14:104776689 (GRCh38)<br>14:105243026 (GRCh37)	C to T
123	rs774284624	14:104775133 (GRCh38)<br>14:105241470 (GRCh37)	C to A or T
124	rs774360915	14:104774970 (GRCh38)<br>14:105241307 (GRCh37)	C to T
125	rs774414969	14:104772416 (GRCh38)<br>14:105238753 (GRCh37)	C to T
126	rs774745066	14:104775764 (GRCh38)<br>14:105242101 (GRCh37)	T to C or G
127	rs775612804	14:104773934 (GRCh38)<br>14:105240271 (GRCh37)	A to G
128	rs775892860	14:104776741 (GRCh38)<br>14:105243078 (GRCh37)	G to A
129	rs776345122	14:104775738 (GRCh38)<br>14:105242075 (GRCh37)	C to T
130	rs777696700	14:104772382 (GRCh38)<br>14:105238719 (GRCh37)	G to A
131	rs778838358	14:104773914 (GRCh38)<br>14:105240251 (GRCh37)	C to T
132	rs779874420	14:104773547 (GRCh38)<br>14:105239884 (GRCh37)	A to C
133	rs780173607	14:104780171 (GRCh38)<br>14:105246508 (GRCh37)	T to C
134	rs781232725	14:104773920 (GRCh38)<br>14:105240257 (GRCh37)	C to T
135	rs781749630	14:104773077 (GRCh38)<br>14:105239414 (GRCh37)	C to T
136	rs866169013	14:104775167 (GRCh38)<br>14:105241504 (GRCh37)	C to A
137	rs889182971	14:104772932 (GRCh38)<br>14:105239269 (GRCh37)	C to T
138	rs897360247	14:104776659 (GRCh38)<br>14:105242996 (GRCh37)	C to T
139	rs908575682	14:104775750 (GRCh38)<br>14:105242087 (GRCh37)	G to C
140	rs913653954	14:104770846 (GRCh38)<br>14:105237183 (GRCh37)	A to G
141	rs923841704	14:104776767 (GRCh38)<br>14:105243104 (GRCh37)	C to T
142	rs937869519	14:104772390 (GRCh38)<br>14:105238727 (GRCh37)	A to C
143	rs941063820	14:104774955 (GRCh38)<br>14:105241292 (GRCh37)	T to C
144	rs955764429	14:104773345 (GRCh38)<br>14:105239682 (GRCh37)	A to G
145	rs971568277	14:104773963 (GRCh38)<br>14:105240300 (GRCh37)	G to C or T

146	rs976500042	14:104772999 (GRCh38)<br>14:105239336 (GRCh37)	T to C
147	rs980441042	14:104775165 (GRCh38)<br>14:105241502 (GRCh37)	T to C
148	rs990046031	14:104780181 (GRCh38)<br>14:105246518 (GRCh37)	G to A
149	rs1012676649	14:104776720 (GRCh38)<br>14:105243057 (GRCh37)	G to A
150	rs1016707349	14:104775132 (GRCh38)<br>14:105241469 (GRCh37)	C to A or T
151	rs1038322721	14:104775774 (GRCh38)<br>14:105242111 (GRCh37)	T to C
152	rs1050565251	14:104772924 (GRCh38)<br>14:105239261 (GRCh37)	C to A
153	rs1163499100	14:104773979 (GRCh38)<br>14:105240316 (GRCh37)	G to A or C
154	rs1165092690	14:104773544 (GRCh38)<br>14:105239881 (GRCh37)	C to T
155	rs1166659979	14:104775654 (GRCh38)<br>14:105241991 (GRCh37)	C to T
156	rs1167819752	14:104773478 (GRCh38)<br>14:105239815 (GRCh37)	T to C
157	rs1168658858	14:104773014 (GRCh38)<br>14:105239351 (GRCh37)	G to A
158	rs1170677405	14:104772998 (GRCh38)<br>14:105239335 (GRCh37)	T to C
159	rs1171160211	14:104773071 (GRCh38)<br>14:105239408 (GRCh37)	C to T
160	rs1173631887	14:104792621 (GRCh38)<br>14:105258958 (GRCh37)	T to C
161	rs1176264024	14:104774973 (GRCh38)<br>14:105241310 (GRCh37)	G to A
162	rs1183083759	14:104775141 (GRCh38)<br>14:105241478 (GRCh37)	T to C
163	rs1184173073	14:104780189 (GRCh38)<br>14:105246526 (GRCh37)	C to T
164	rs1197941200	14:104773043 (GRCh38)<br>14:105239380 (GRCh37)	C to T
165	rs1200003171	14:104775122 (GRCh38)<br>14:105241459 (GRCh37)	C to A or T
166	rs1209929395	14:104773505 (GRCh38)<br>14:105239842 (GRCh37)	C to T
167	rs1216778719	14:104775084 (GRCh38)<br>14:105241421 (GRCh37)	C to T
168	rs1219649544	14:104773013 (GRCh38)<br>14:105239350 (GRCh37)	C to T
169	rs1223729648	14:104780151 (GRCh38)<br>14:105246488 (GRCh37)	A to G
170	rs1230091567	14:104775685 (GRCh38)<br>14:105242022 (GRCh37)	C to A
171	rs1235749501	14:104792607 (GRCh38)<br>14:105258944 (GRCh37)	G to A
172	rs1240566715	14:104774957 (GRCh38)<br>14:105241294 (GRCh37)	G to A or C
173	rs1244026143	14:104775152 (GRCh38)<br>14:105241489 (GRCh37)	A to C
174	rs1244832277	14:104773561 (GRCh38)<br>14:105239898 (GRCh37)	C to T
175	rs1247419183	14:104773041 (GRCh38)	C to T

		14:105239378 (GRCh37)	
176	rs1268659696	14:104775736 (GRCh38)<br>14:105242073 (GRCh37)	C to A or T
177	rs1274533572	14:104773070 (GRCh38)<br>14:105239407 (GRCh37)	C to A
178	rs1276744835	14:104773930 (GRCh38)<br>14:105240267 (GRCh37)	C to G or T
179	rs1277434432	14:104775202 (GRCh38)<br>14:105241539 (GRCh37)	C to T
180	rs1281415838	14:104775780 (GRCh38)<br>14:105242117 (GRCh37)	T to C
181	rs1295342651	14:104772414 (GRCh38)<br>14:105238751 (GRCh37)	T to C
182	rs1295857274	14:104772385 (GRCh38)<br>14:105238722 (GRCh37)	G to T
183	rs1296782302	14:104776668 (GRCh38)<br>14:105243005 (GRCh37)	G to C or T
184	rs1297345295	14:104773553 (GRCh38)<br>14:105239890 (GRCh37)	C to T
185	rs1298334491	14:104775785 (GRCh38)<br>14:105242122 (GRCh37)	G to A
186	rs1301434623	14:104780132 (GRCh38)<br>14:105246469 (GRCh37)	T to G
187	rs1302148730	14:104772900 (GRCh38)<br>14:105239237 (GRCh37)	G to A
188	rs1308190883	14:104772379 (GRCh38)<br>14:105238716 (GRCh37)	C to T
189	rs1310418829	14:104773558 (GRCh38)<br>14:105239895 (GRCh37)	T to C
190	rs1316268769	14:104776738 (GRCh38)<br>14:105243075 (GRCh37)	G to C
191	rs1319030326	14:104775693 (GRCh38)<br>14:105242030 (GRCh37)	C to T
192	rs1324208253	14:104775678 (GRCh38)<br>14:105242015 (GRCh37)	A to C
193	rs1335182846	14:104772984 (GRCh38)<br>14:105239321 (GRCh37)	T to G
194	rs1338237897	14:104792630 (GRCh38)<br>14:105258967 (GRCh37)	G to C
195	rs1338783398	14:104775744 (GRCh38)<br>14:105242081 (GRCh37)	C to T
196	rs1339827175	14:104773559 (GRCh38)<br>14:105239896 (GRCh37)	C to T
197	rs1340200537	14:104773029 (GRCh38)<br>14:105239366 (GRCh37)	C to T
198	rs1340970650	14:104773556 (GRCh38)<br>14:105239893 (GRCh37)	G to A
199	rs1342613654	14:104772426 (GRCh38)<br>14:105238763 (GRCh37)	T to C or G
200	rs1352687393	14:104775155 (GRCh38)<br>14:105241492 (GRCh37)	T to A or C
201	rs1358103245	14:104780117 (GRCh38)<br>14:105246454 (GRCh37)	T to C
202	rs1359340183	14:104772957 (GRCh38)<br>14:105239294 (GRCh37)	C to T
203	rs1360782672	14:104770412 (GRCh38)<br>14:105236749 (GRCh37)	T to C
204	rs1367895795	14:104773926 (GRCh38)<br>14:105240263 (GRCh37)	C to T

205	rs1369639631	14:104770833 (GRCh38)<br>14:105237170 (GRCh37)	G to T
206	rs1371607114	14:104775206 (GRCh38)<br>14:105241543 (GRCh37)	G to A
207	rs1373202839	14:104770801 (GRCh38)<br>14:105237138 (GRCh37)	C to T
208	rs1373983688	14:104772396 (GRCh38)<br>14:105238733 (GRCh37)	C to G or T
209	rs1377831067	14:104773297 (GRCh38)<br>14:105239634 (GRCh37)	G to A
210	rs1380514442	14:104780205 (GRCh38)<br>14:105246542 (GRCh37)	T to C
211	rs1390179436	14:104773475 (GRCh38)<br>14:105239812 (GRCh37)	C to T
212	rs1390801321	14:104772884 (GRCh38)<br>14:105239221 (GRCh37)	T to C
213	rs1394191738	14:104772879 (GRCh38)<br>14:105239216 (GRCh37)	T to C
214	rs1396308032	14:104776746 (GRCh38)<br>14:105243083 (GRCh37)	C to T
215	rs1397852501	14:104773490 (GRCh38)<br>14:105239827 (GRCh37)	G to A
216	rs1404637346	14:104792627 (GRCh38)<br>14:105258964 (GRCh37)	A to G
217	rs1418994529	14:104770388 (GRCh38)<br>14:105236725 (GRCh37)	T to A
218	rs1424916218	14:104773465 (GRCh38)<br>14:105239802 (GRCh37)	C to T
219	rs1431676094	14:104770408 (GRCh38)<br>14:105236745 (GRCh37)	T to C
220	rs1438024792	14:104772986 (GRCh38)<br>14:105239323 (GRCh37)	T to C
221	rs1444891733	14:104772391 (GRCh38)<br>14:105238728 (GRCh37)	C to T
222	rs1445658903	14:104775671 (GRCh38)<br>14:105242008 (GRCh37)	G to A
223	rs1454909926	14:104773088 (GRCh38)<br>14:105239425 (GRCh37)	A to G
224	rs1462219795	14:104774972 (GRCh38)<br>14:105241309 (GRCh37)	C to T
225	rs1467510263	14:104770357 (GRCh38)<br>14:105236694 (GRCh37)	G to A
226	rs1468001776	14:104772376 (GRCh38)<br>14:105238713 (GRCh37)	A to C
227	rs1476861882	14:104780109 (GRCh38)<br>14:105246446 (GRCh37)	G to A
228	rs1480573852	14:104772920 (GRCh38)<br>14:105239257 (GRCh37)	T to C
229	rs1566816661	14:104773479 (GRCh38)<br>14:105239816 (GRCh37)	C to G
230	rs1566818045	14:104775672 (GRCh38)<br>14:105242009 (GRCh37)	C to G
231	rs1566818890	14:104776693 (GRCh38)<br>14:105243030 (GRCh37)	C to T
232	rs1566818959	14:104776757 (GRCh38)<br>14:105243094 (GRCh37)	C to T
233	rs1595239049	14:104770766 (GRCh38)<br>14:105237103 (GRCh37)	T to G
234	rs1595242014	14:104773040 (GRCh38)	A to C

		14:105239377 (GRCh37)	
235	rs1595244672	14:104774982 (GRCh38)<br>14:105241319 (GRCh37)	T to G
236	rs1595245738	14:104775741 (GRCh38)<br>14:105242078 (GRCh37)	C to T
237	rs142646145	14:104776679 (GRCh38)<br>14:105243016 (GRCh37)	A to C<br>A to G
238	rs1595251483	14:104780213 (GRCh38)<br>14:105246550 (GRCh37)	TC to CT (It was ignored because it is MNV not SNP)
239	rs1892340313	14:104770757 (GRCh38)<br>14:105237094 (GRCh37)	G to A
240	rs1892344387	14:104770831 (GRCh38)<br>14:105237168 (GRCh37)	T to G
241	rs1892477463	14:104772953 (GRCh38)<br>14:105239290 (GRCh37)	A to G
242	rs1892478123	14:104772962 (GRCh38)<br>14:105239299 (GRCh37)	A to G
243	rs1892486033	14:104773067 (GRCh38)<br>14:105239404 (GRCh37)	C to T
244	rs1892503990	14:104773292 (GRCh38)<br>14:105239629 (GRCh37)	T to C
245	rs1892518022	14:104773484 (GRCh38)<br>14:105239821 (GRCh37)	C to T
246	rs1892518764	14:104773487 (GRCh38)<br>14:105239824 (GRCh37)	A to C
247	rs1892523027	14:104773534 (GRCh38)<br>14:105239871 (GRCh37)	G to A
248	rs1892659749	14:104775710 (GRCh38)<br>14:105242047 (GRCh37)	C to G
249	rs1892954317	14:104780195 (GRCh38)<br>14:105246532 (GRCh37)	C to T
250	rs1893685625	14:104792628 (GRCh38)<br>14:105258965 (GRCh37)	T to C
251	rs1264566007	14:104775703 (GRCh38)<br>14:105242040 (GRCh37)	G to A or T
252	rs1770844018	14:104773289 (GRCh38)<br>14:105239626 (GRCh37)	T to C
253	rs1883198727	14:104772917 (GRCh38)<br>14:105239254 (GRCh37)	G to C
254	rs1892308596	14:104770354 (GRCh38)<br>14:105236691 (GRCh37)	C to T
255	rs1892342047	14:104770786 (GRCh38)<br>14:105237123 (GRCh37)	T to G
256	rs1892342488	14:104770791 (GRCh38)<br>14:105237128 (GRCh37)	A to C
257	rs1892447426	14:104772442 (GRCh38)<br>14:105238779 (GRCh37)	C to T
258	rs1892447721	14:104772445 (GRCh38)<br>14:105238782 (GRCh37)	C to T
259	rs1892472772	14:104772896 (GRCh38)<br>14:105239233 (GRCh37)	T to C
260	rs1892473652	14:104772913 (GRCh38)<br>14:105239250 (GRCh37)	C to G
261	rs1892475655	14:104772939 (GRCh38)<br>14:105239276 (GRCh37)	T to C
262	rs1892481082	14:104772992 (GRCh38)<br>14:105239329 (GRCh37)	T to C
263	rs1892483378	14:104773017 (GRCh38)<br>14:105239354 (GRCh37)	C to T

264	rs1892502921	14:104773273 (GRCh38)<br>14:105239610 (GRCh37)	G to A
265	rs1892504590	14:104773309 (GRCh38)<br>14:105239646 (GRCh37)	A to T
266	rs1892504858	14:104773318 (GRCh38)<br>14:105239655 (GRCh37)	T to C
267	rs1892506436	14:104773352 (GRCh38)<br>14:105239689 (GRCh37)	C to T
268	rs1892507073	14:104773370 (GRCh38)<br>14:105239707 (GRCh37)	G to A
269	rs1892520149	14:104773498 (GRCh38)<br>14:105239835 (GRCh37)	T to C
270	rs1892520597	14:104773501 (GRCh38)<br>14:105239838 (GRCh37)	A to G
271	rs1892525199	14:104773555 (GRCh38)<br>14:105239892 (GRCh37)	C to T
272	rs1892528134	14:104773576 (GRCh38)<br>14:105239913 (GRCh37)	A to G
273	rs1892553720	14:104773938 (GRCh38)<br>14:105240275 (GRCh37)	C to G
274	rs1892621826	14:104774978 (GRCh38)<br>14:105241315 (GRCh37)	T to C
275	rs1892626748	14:104775077 (GRCh38)<br>14:105241414 (GRCh37)	T to G
276	rs1892631024	14:104775185 (GRCh38)<br>14:105241522 (GRCh37)	A to G
277	rs1892631592	14:104775198 (GRCh38)<br>14:105241535 (GRCh37)	C to T
278	rs1892658092	14:104775666 (GRCh38)<br>14:105242003 (GRCh37)	G to A
279	rs1892658609	14:104775680 (GRCh38)<br>14:105242017 (GRCh37)	A to G
280	rs1892659463	14:104775701 (GRCh38)<br>14:105242038 (GRCh37)	G to A
281	rs1892660002	14:104775719 (GRCh38)<br>14:105242056 (GRCh37)	C to T
282	rs1892663954	14:104775759 (GRCh38)<br>14:105242096 (GRCh37)	G to A
283	rs1892665030	14:104775767 (GRCh38)<br>14:105242104 (GRCh37)	G to T
284	rs1892666135	14:104775782 (GRCh38)<br>14:105242119 (GRCh37)	G to A
285	rs1892666993	14:104775788 (GRCh38)<br>14:105242125 (GRCh37)	G to A
286	rs1892732634	14:104776666 (GRCh38)<br>14:105243003 (GRCh37)	C to T
287	rs1892738131	14:104776764 (GRCh38)<br>14:105243101 (GRCh37)	T to A
288	rs1892948595	14:104780090 (GRCh38)<br>14:105246427 (GRCh37)	G to A
289	rs1892948718	14:104780091 (GRCh38)<br>14:105246428 (GRCh37)	C to A
290	rs1892951094	14:104780138 (GRCh38)<br>14:105246475 (GRCh37)	G to A
291	rs1892954511	14:104780196 (GRCh38)<br>14:105246533 (GRCh37)	G to A or T
292	rs1892954980	14:104780201 (GRCh38)<br>14:105246538 (GRCh37)	G to A
293	rs1893686640	14:104792639 (GRCh38)	C to T

		14:105258976 (GRCh37)	
