# Supplementary material for: Discovering Deleterious Single Nucleotide Polymorphisms of Human AKT1 Oncogene: An In Silico Study
Source: Life (Basel). 2023 Jul 10;13(7):1532. doi: 10.3390/life13071532 (PMC10381612; doi:10.3390/life13071532)
Supplement: Supplementary file 1 [file life-13-01532-s001.zip › Supplementary Table S2.pdf]

Supplementary Table S2: Prediction of the functional effect of SNPs by different servers. (B=Benign; DG=Damaging; DL= Deleterious; D= Disease; E= Effect; H= High; L=Low; M=Medium; MA=Mutation assessor; N=Neutral, P=Pathogenic; PSD: Possibly Damaging; PB= Probably benign; PD= Probably damaging; S=score; TL=Tolerated and U=Unknown)

dbSNP ID	Substitution	PROVEAN	S	SIFT	S	POLYPHEN-2	S	SNAP-2	S	MA	S	PANTHER	PON-P2	S	P-MUT	S
rs113547523	Arg465His	N	-1.14	TL	0.073	B	0.008	N	-52	L	1.355	PB	U	0.715	N	0.20
rs121434592	Glu17Lys	DL	-3.68	DG	0.004	PD	1.000	E	7	M	2.47	PB	U	0.794	D	0.67
rs140532443	Asp3Asn	N	-1.39	DG	0.024	B	0.002	N	-88	L	0.895	PB	U	0.419	N	0.22
rs144112075	Ser463Arg	N	-0.88	TL	0.317	B	0.002	N	-43	L	1.15	PB	P	0.777	N	0.43
rs146483593	Gly410Ser	N	1.48	TL	1.000	B	0.000	N	-88	N	-0.25	PB	U	0.433	N	0.27
rs146875699	Asp46Glu	N	-1.34	TL	0.223	B	0.055	N	-73	L	0.94	PB	U	0.547	N	0.34
rs183989506	Arg69Gln	N	-1.87	TL	0.089	B	0.036	E	40	M	2.195	PB	U	0.628	D	0.6
rs188580689	Ala399Thr	DL	-3.35	TL	0.094	PD	1.000	E	25	L	1.525	PB	U	0.772	N	0.43
rs374093099	Arg48Cys	N	-1.55	TL	0.078	PSD	0.626	N	-58	L	1.04	PB	P	0.857	N	0.26
rs375395037	Ala255Thr	DL	-3.74	DG	0.000	PD	1.000	N	-6	L	1.195	PD	U	0.803	D	0.79
rs375990114	Arg144Cys	DL	-3.81	DG	0.007	B	0.002	E	61	N	0.345	PB	U	0.662	N	0.34
rs397514644	Arg25Cys	DL	-7.22	DG	0.000	PD	1.000	E	76	H	3.93	PB	U	0.824	D	0.81
rs397514645	Thr435Pro	DL	-5.80	DG	0.009	PD	1.000	E	63	H	3.65	PB	U	0.701	D	0.76
rs549083521	Ser266Leu	DL	-3.20	TL	0.071	PSD	0.896	N	-21	M	2.825	PB	P	0.826	D	0.61
rs587778018	Met458Thr	DL	-2.62	TL	0.080	B	0.003	N	-43	M	1.955	PB	P	0.823	N	0.5
rs587778019	Ile75Met	N	-2.00	DG	0.016	PD	0.999	E	2	M	3.155	PB	P	0.819	D	0.51
rs745803788	Glu464Lys	DL	-2.71	DG	0.007	PSD	0.551	N	-16	L	1.79	PB	U	0.814	N	0.49
rs746272761	Arg370His	DL	-3.35	TL	0.123	PD	0.975	N	-38	L	1.24	PB	U	0.706	N	0.29
rs754031503	Val4Leu	N	-0.83	TL	0.200	B	0.002	N	-94	N	0.73	PB	U	0.710	N	0.46
rs758157217	Ile36Thr	DL	-3.46	TL	0.069	PSD	0.949	N	-11	L	1.77	PB	U	0.664	N	0.49
rs758456890	Met147Thr	DL	-3.93	DG	0.002	B	0.377	E	55	L	1.935	PB	U	0.784	D	0.74

rs759702315	Gln454Arg	N	-1.11	TL	0.642	B	0.000	N	-80	N	-0.215	PB	U	0.752	N	0.27
rs762705090	Arg367Cys	DL	-6.49	DG	0.001	PD	1.000	E	7	M	1.98	PB	P	0.879	D	0.61
rs764863282	Glu418Lys	N	-1.78	TL	0.364	B	0.010	N	-73	N	0.55	PB	U	0.802	N	0.33
rs768070795	Ala171Val	DL	-2.83	DG	0.047	PSD	0.598	N	-86	N	0.42	PB	P	0.791	N	0.34
rs768606668	Arg241Trp	DL	-6.24	DG	0.040	PSD	0.877	E	52	M	2.81	PB	P	0.894	D	0.75
rs771065764	Arg76His	DL	-4.41	DG	0.001	PD	1.000	E	39	M	2.705	PB	U	0.798	D	0.77
rs773607483	Lys400Glu	N	-2.42	TL	0.303	B	0.005	N	-49	N	0.29	PB	U	0.692	N	0.16
rs774836044	Arg48His	N	0.48	TL	0.272	B	0.000	N	-69	N	0.695	PB	U	0.735	N	0.19
rs778376616	Val136Met	N	-0.36	TL	0.255	B	0.061	N	-67	L	1.245	PB	P	0.763	N	0.36
rs780207480	Asp190Glu	DL	-3.70	TL	0.129	B	0.112	N	-59	N	0.175	PB	U	0.736	N	0.31
rs780571834	Arg251Cys	DL	-6.40	DG	0.013	PD	0.992	E	3	L	1.76	PB	P	0.903	N	0.28
rs781339141	Ile36Val	N	-0.59	TL	0.318	B	0.198	N	-50	L	1.14	PB	U	0.638	N	0.4
rs781388586	Thr371Met	DL	-3.04	TL	0.279	PSD	0.606	N	-50	L	1.335	PB	U	0.728	N	0.34
rs983007851	Arg174Cys	DL	-3.83	DG	0.011	PSD	0.900	N	-35	L	1.91	PB	P	0.868	N	0.28
rs1057518602	Gln79Arg	DL	-3.53	DG	0.004	PD	0.998	N	-2	M	2.21	PB	U	0.738	D	0.5
rs1057519804	Gln79Lys	DL	-3.56	DG	0.023	PD	0.996	E	11	M	2.25	PB	P	0.814	D	0.6
rs1060503071	Arg144His	DL	-2.59	DG	0.003	PSD	0.505	E	61	N	0.345	PB	U	0.633	N	0.23
rs1159942120	Glu440Asp	N	-1.27	TL	0.462	B	0.000	N	-94	N	0.49	PB	P	0.782	N	0.36
rs1205616929	Gly478Ser	N	-0.14	TL	0.223	PSD	0.924	N	-66	N	0.465	PB	U	0.793	N	0.49
rs1334042967	Glu397Asp	N	0.74	TL	1.000	B	0	N	-89	N	0.76	PB	U	0.394	N	0.24
rs1360111387	Gly303Ser	DL	-4.92	TL	0.134	B	0.063	N	-14	N	0.765	PB	U	0.705	N	0.34
rs1457484217	Arg121Gln	N	0.68	TL	0.633	B	0.013	N	-53	N	0.69	PB	U	0.565	N	0.17
rs1458420660	Arg249Trp	DL	-7.11	DG	0.000	PD	1.000	E	64	L	1.025	PB	P	0.904	D	0.61
rs1555383354	Gln414His	DL	-2.73	DG	0.017	B	0.275	N	-92	M	2.29	PB	P	0.764	N	0.45
rs1555383471	Met306Thr	N	1.36	TL	1.000	B	0.001	N	-53	N	-0.88	PB	P	0.869	N	0.17
rs1555383511	Ile257Thr	DL	-4.66	DG	0.000	PD	1.000	E	7	M	2.195	PD	U	0.821	D	0.90
rs1555383695	Lys182Arg	N	-1.79	TL	0.322	B	0.028	N	-69	L	1.455	PB	U	0.640	N	0.18

rs1566815164	Leu421Phe	DL	-2.85	DG	0.002	PSD	0.848	E	4	M	3.25	PB	P	0.801	D	0.76
rs1566816289	Asp387Asn	DL	-3.56	TL	0.110	B	0.196	N	-72	N	0.575	PB	P	0.811	N	0.11
rs1566818099	Asp119Gly	N	-2.12	TL	0.125	B	0.005	N	-25	L	1.385	PB	U	0.737	D	0.6
rs1566826869	Ser2Gly	N	-1.03	DG	0.024	B	0.005	N	-96	L	1.93	PB	U	0.456	N	0.32
rs1595243377	Asn231Ser	DL	-4.39	DG	0.002	PD	1.00	N	-48	N	-0.085	PB	P	0.816	N	0.32
rs11555431	Pro388Thr	DL	-7.59	DG	0.002	PD	0.999	E	23	M	3.235	PB	U	0.733	D	0.68
rs11555432	Leu357Pro	DL	-6.77	DG	0.000	PD	1.000	E	67	M	2.91	PB	P	0.897	D	0.9
rs11555433	Val167Ala	N	-0.87	TL	0.861	B	0.039	N	-65	N	-0.4	PB	P	0.837	N	0.16
rs11555435	Val461Leu	N	-0.81	TL	0.745	B	0.000	N	-92	N	-0.48	PB	U	0.644	N	0.33
rs12881616	Glu319Gly	DL	-6.76	DG	0.001	PD	0.999	E	60	H	4.065	PD	U	0.724	D	0.90
rs142843688	His143Tyr	N	-1.77	DG	0.032	B	0.001	N	-10	N	0.69	PB	P	0.778	D	0.71
rs143266084	Arg406His	N	-1.21	TL	0.128	B	0.001	E	19	L	1.675	PB	U	0.579	N	0.25
rs144128670	Ala188Thr	N	-2.14	TL	0.217	B	0.051	N	-77	N	0.42	PB	U	0.717	N	0.48
rs201291259	Thr479Met	N	-1.42	DG	0.002	PD	0.991	N	0	L	1.495	PB	U	0.748	N	0.25
rs201636005	Asp32Glu	DL	-3.37	DG	0.025	PD	0.999	E	48	M	2.78	PB	U	0.735	D	0.58
rs368797346	Arg15Gln	DL	-2.83	TL	0.161	PSD	0.538	E	37	L	1.325	PB	U	0.448	N	0.44
rs369198922	Arg121Trp	N	-2.10	DG	0.014	PD	0.995	E	31	N	0.69	PB	U	0.692	N	0.63
rs369520527	Arg367His	DL	-3.97	DG	0.005	PD	0.989	E	5	L	1.43	PB	U	0.734	N	0.18
rs369698909	Arg222His	DL	-3.50	TL	0.165	B	0.396	N	-61	N	-0.365	PB	p	0.822	N	0.07
rs371467719	Asp262Glu	N	-0.58	TL	0.449	B	0.001	N	-65	N	-0.27	PB	U	0.749	N	0.07
rs372502847	Met363Val	N	-2.03	TL	0.641	B	0.008	N	-69	N	0.345	PB	U	0.666	N	0.23
rs373253729	Glu375Lys	DL	-2.77	TL	0.075	PSD	0.890	E	42	L	1.79	PB	P	0.830	N	0.32
rs531850885	Glu397Lys	N	-2.01	TL	0.136	B	0.001	N	-71	N	0.19	PB	P	0.783	N	0.37
rs532268608	Gln47His	N	-0.81	TL	0.547	B	0.003	N	-84	N	-0.05	PB	U	0.703	N	0.20
rs549370342	Arg370Ser	DL	-2.99	TL	0.667	B	0.033	N	-26	N	-0.285	PB	U	0.727	N	0.27
rs551254461	Arg222Cys	DL	-6.29	TL	0.060	PSD	0.898	E	31	N	0.375	PB	P	0.905	N	0.49

rs568870136	Gly157Arg	DL	-7.48	DG	0.001	PD	1.000	E	91	H	4.01	PD	P	0.852	D	0.90
rs745500951	Val185Gly	DL	-6.51	DG	0.001	PD	0.983	E	25	N	0.49	PB	U	0.658	D	0.90
rs745809388	Arg249Gln	DL	-3.50	DG	0.000	PD	1.000	E	3	N	-0.77	PB	P	0.902	N	0.29
rs746397537	Asn324Ser	DL	-3.43	TL	0.009	B	0.288	N	-28	N	-0.215	PB	P	0.856	N	0.18
rs746934495	Ser122Leu	N	-1.52	TL	0.636	B	0.001	N	-47	N	0.69	PB	U	0.783	D	0.63
rs748789094	Thr172Ile	DL	-4.87	DG	0.002	PSD	0.951	E	34	M	2.79	PB	U	0.710	D	0.72
rs749186394	Thr430Met	DL	-4.00	DG	0.044	PSD	0.822	N	-67	M	2.685	PB	U	0.799	N	0.36
rs749544983	Arg406Cys	DL	2.91	DG	0.022	PD	0.957	E	38	L	1.815	PB	P	0.803	N	0.30
rs750653493	Thr34Asn	N	-1.41	DG	0.044	B	0.025	N	-36	L	1.135	PB	U	0.732	N	0.35
rs751232562	Tyr253Cys	DL	-8.07	DG	0.006	PD	1.000	N	-4	M	2.71	PD	P	0.863	D	0.90
rs751243134	Thr65Met	N	-2.49	TL	0.064	PD	0.996	N	-57	M	3.12	PB	U	0.806	D	0.89
rs751416672	Leu196His	DL	-5.45	DG	0.001	PD	0.997	E	33	L	1.355	PB	P	0.876	N	0.25
rs751976958	Met446Ile	N	-1.16	TL	0.091	B	0.002	N	-72	N	0.345	PB	U	0.730	N	0.30
rs753190812	Pro452His	DL	-7.95	DG	0.000	PD	1.000	E	29	H	3.57	PD	U	0.807	D	0.77
rs753360468	Lys420Glu	DL	-3.40	TL	0.236	B	0.100	N	-27	L	1.815	PB	P	0.825	N	0.22
rs753765116	Arg41Trp	DL	-4.62	TL	0.072	B	0.126	E	52	M	2.345	PB	P	0.860	D	0.83
rs754040453	Asp455Gly	N	-0.49	TL	0.410	B	0.002	N	-51	N	0.49	PB	U	0.672	N	0.46
rs754500025	Lys419Arg	N	-2.08	TL	0.151	B	0.006	N	-95	N	0.775	PB	U	0.629	N	0.12
rs755597789	Ser431Leu	DL	-5.51	DG	0.002	PD	0.999	N	-2	H	3.635	PB	P	0.873	D	0.89D
rs756293014	Ala193Val	DL	-3.62	DG	0.038	PSD	0.569	N	-77	N	0.64	PB	P	0.791	D	0.61
rs756697784	Arg251His	DL	-4.21	DG	0.001	PD	1.000	E	11	L	1.875	PB	P	0.821	D	0.89
rs757963528	Tyr474Ser	DL	-6.58	TL	0.052	PSD	0.810	E	76	M	3.305	PB	U	0.558	D	0.94
rs758025607	Leu261Val	DL	-2.77	DG	0.035	PSD	0.869	N	-57	L	1.895	PB	U	0.740	N	0.29
rs758476416	His415Gln	N	0.77	TL	0.052	B	0.003	N	-83	N	-0.975	PB	U	0.615	N	0.005
rs759031755	Pro125Leu	N	-1.94	TL	0.083	B	0.012	E	7	L	1.79	PB	P	0.892	D	0.63

rs759902535	Ser457Arg	N	-2.02	TL	0.058	B	0.199	N	-70	M	2.02	PB	U	0.647	D	0.53
rs760044481	Thr72Ala	DL	-3.85	TL	0.105	B	0.080	N	-51	L	1.125	PB	P	0.851	D	0.66
rs760384228	Tyr215Cys	DL	-7.00	DG	0.037	PSD	0.777	E	19	M	1.99	PB	P	0.873	D	0.53
rs760536822	Arg96Trp	DL	-6.62	DG	0.042	PSD	0.939	E	40	H	3.73	PB	P	0.863	D	0.83
rs761151169	Thr101Ala	N	-0.27	TL	0.513	B	0.000	N	-73	N	-0.41	PB	U	0.615	N	0.18
rs762040581	Asp221His	DL	-6.16	DG	0.009	PD	1.000	N	-6	N	0.63	PB	P	0.859	N	0.25
rs762559261	Arg67Trp	DL	-6.20	DG	0.024	PD	0.999	E	72	M	2.24	PB	P	0.872	D	0.88
rs764514218	Ile447Val	N	0.26	TL	1.000	B	0.001	N	-81	L	1.145	PB	U	0.762	N	0.34
rs764931115	Lys39Glu	DL	-3.47	DG	0.001	PD	1.000	E	82	M	2.885	PB	P	0.813	D	0.52
rs765264778	Asp453Glu	N	-1.75	TL	0.249	B	0.000	N	-83	L	1.04	PB	U	0.688	D	0.66
rs766000895	Arg41Gln	N	-1.30	TL	0.109	B	0.253	N	-32	N	0.675	PB	P	0.852	N	0.41
rs766546254	Ala5Thr	N	0.23	TL	0.782	B	0.000	N	-95	N	-0.55	PB	U	0.467	N	0.11
rs766798551	Glu397Ala	DL	-3.15	DG	0.043	B	0.002	N	-78	N	- 0.045	PB	U	0.694	N	0.43
rs767699129	Ile288Val	N	-0.83	TL	0.133	B	0.030	N	-84	N	0.59	PD	U	0.490	N	0.18
rs768660759	His405Pro	DL	-9.44	DG	0.001	PSD	0.790	E	86	H	3.96	PD	P	0.878	D	0.90
rs768800433	Gly109Ser	N	1.56	TL	0.897	B	0.001	N	-91	N	- 0.235	PB	U	0.461	N	0.27
rs768898540	Ala50Val	N	-0.68	TL	0.296	B	0.011	N	-95	N	0.505	PB	U	0.509	N	0.21
rs769619023	Met178Thr	DL	-3.40	DG	0.002	PD	0.996	E	61	M	1.965	PB	U	0.775	D	0.90
rs770370100	Arg465Cys	DL	-2.63	DG	0.032	PD	0.995	E	4	L	1.7	PB	P	0.894	N	0.39
rs770980034	Leu235Val	N	-2.47	DG	0.004	PSD	0.885	E	16	M	2.715	PD	U	0.740	N	0.29
rs772473246	Arg86Cys	DL	-7.15	DG	0.000	PD	1.000	E	52	M	3.47	PB	P	0.851	D	0.86
rs773520823	Ser240Cys	DL	-4.39	DG	0.001	PD	1.00	N	-53	M	1.965	PB	P	0.818	D	0.61
rs773971502	Arg86His	DL	-4.14	TL	0.054	PD	0.987	N	-10	M	3.215	PB	P	0.981	D	0.72
rs774284624	Lys170Asn	DL	-4.37	DG	0.020	PSD	0.800	E	26	L	1.095	PB	U	0.738	N	0.47
rs774360915	Val201Ile	N	-0.88	TL	0.152	B	0.398	N	-61	N	-1.29	PB	U	0.738	N	0.09
rs774414969	Met403Ile	DL	-3.36	DG	0.012	PSD	0.818	N	-34	N	0.235	PB	P	0.841	D	0.61

rs774745066	Asp108Ala	DL	-3.41	TL	0.333	B	0.001	N	-46	L	1.355	PB	U	0.656	D	0.62
rs775612804	Met227Thr	DL	-5.63	DG	0.000	PD	1.000	E	59	L	1.535	PB	P	0.900	N	0.32
rs775892860	Arg69Trp	DL	-5.54	DG	0.001	PD	1.000	E	59	M	3.09	PB	P	0.771	D	0.89
rs776345122	Glu117Lys	N	-0.12	TL	0.762	B	0.000	N	-76	N	0	PB	U	0.626	N	0.22
rs777696700	His415Tyr	N	-1.84	DG	0.002	B	0.000	N	-52	N	0	PB	U	0.651	N	0.26
rs778838358	Glu234Lys	DL	-3.77	DG	0.001	PD	1.000	E	81	M	2.05	PB	P	0.885	D	0.90
rs779874420	Ser246Ala	N	-1.80	TL	0.182	B	0.095	N	-10	L	1.63	PB	U	0.698	N	0.28
rs780173607	Asn31Ser	N	0.49	TL	1.000	B	0.000	N	-96	N	-1.405	PB	U	0.423	N	0.10
rs781232725	Gly232Arg	DL	-7.15	DG	0.032	PD	1.000	E	63	M	2.445	PB	P	0.919	D	0.9
rs781749630	Asp325Asn	DL	-4.37	TL	0.079	B	0.360	E	16	N	0.59	PB	P	0.820	N	0.25
rs866169013	Gly159Val	DL	-8.37	DG	0.000	PD	0.967	E	90	H	4.815	PD	P	0.861	D	0.90
rs889182971	Gly373Asp	N	-0.89	DG	0.002	B	0.309	E	47	N	-0.03	PB	U	0.650	N	0.23
rs897360247	Arg96Gln	DL	-3.43	DG	0.038	PD	0.995	N	-18	M	2.76	PB	P	0.875	D	0.75
rs908575682	Gln113Glu	N	-0.51	TL	0.517	B	0.001	N	-68	L	1.75	PB	U	0.633	N	0.46
rs913653954	Leu421Pro	DL	-5.43	DG	0.000	PD	0.989	E	61	H	3.595	PB	U	0.843	D	0.80
rs923841704	Cys60Tyr	DL	-8.35	DG	0.003	PD	0.999	E	67	M	2.875	PB	P	0.824	D	0.69
rs937869519	Val412Gly	N	0.38	DG	0.0023	B	0.013	N	-31	L	1.285	PB	U	0.718	N	019
rs941063820	Arg206Gly	DL	-3.19	DG	0.022	B	0.147	E	31	L	1.125	PB	P	0.766	N	0.29
rs955764429	Ile288Thr	DL	-4.68	DG	0.000	PD	1.000	E	23	M	2.495	PD	U	0.733	D	0.90
rs971568277	Phe217Leu	DL	-5.29	DG	0.007	PSD	0.916	E	39	L	1.935	PD	P	0.868	D	0.89
rs976500042	Asn351Asp	DL	-3.52	TL	0.093	PSD	0.710	N	-61	N	-0.02	PB	P	0.759	N	0.09
rs980441042	Thr160Ala	DL	-7.68	DG	0.009	B	0.345	N	-38	N	-0.02	PB	P	0.809	N	0.19
rs990046031	Leu28Phe	N	-2.05	TL	0.051	PSD	0.730	N	-43	L	1.87	PB	P	0.767	N	0.41
rs1012676649	Arg76Cys	DL	-6.65	DG	0.046	PSD	0.939	E	34	H	3.51	PB	P	0.860	D	0.92
rs1016707349	Ala171Ser	N	-1.02	TL	0.451	B	0.001	N	-95	N	0.01	PB	U	0.720	N	0.23
rs1038322721	Thr105Ala	N	-0.05	TL	0.810	B	0.000	N	-78	N	-0.17	PB	U	0.455	N	0.22

rs1050565251	Ala376Ser	DL	-2.64	DG	0.019	PD	0.974	E	1	L	1.45	PD	U	0.690	N	0.25
rs1163499100	Ala212Val	N	-2.26	TL	0.092	B	0.006	N	-86	N	0.67	PB	P	0.805	D	0.81
rs1165092690	Glu247Lys	DL	-3.74	DG	0.002	PD	1.000	E	73	M	2.605	PB	P	0.847	D	0.90
rs1166659979	Val145Met	N	-1.84	DG	0.037	B	0.352	N	-83	L	1.59	PB	U	0.779	N	0.18
rs1167819752	Asn269Asp	N	-1.75	TL	0.452	B	0.001	N	-45	L	1.25	PB	P	0.812	N	0.07
rs1168658858	Arg346Cys	DL	-7.58	DG	0.003	PD	0.001	E	36	L	1.24	PB	P	0.888	D	0.72
rs1170677405	Asn351Ser	DL	-3.24	TL	0.675	B	0.014	N	-86	N	-0.23	PB	U	0.474	N	0.21
rs1171160211	Gly327Ser	DL	-5.85	DG	0.050	PD	1.000	N	-30	N	0.555	PB	P	0.781	N	0.28
rs1173631887	Lys8Arg	N	-1.79	TL	0.086	B	0.209	N	-46	L	1.615	PB	U	0.513	N	0.39
rs1176264024	Arg200Cys	DL	-6.52	TL	0.063	PSD	0.798	N	-4	L	1.67	PB	P	0.815	D	0.88
rs1183083759	Lys168Glu	N	-2.46	DG	0.001	B	0.431	N	-50	N	0.19	PB	P	0.810	N	0.23
rs1184173073	Arg25His	DL	-4.46	DG	0.000	PD	1.000	E	73	H	4.28	PB	U	0.753	D	0.67
rs1197941200	Gly336Asp	DL	-6.84	DG	0.000	PD	1.000	E	89	H	4.64	PD	P	0.895	D	0.90
rs1200003171	Arg174His	N	-0.47	TL	0.216	B	0.001	N	-88	L	1.55	PB	U	0.382	N	0.12
rs1209929395	Ala260Thr	DL	-3.74	DG	0.000	PD	1.000	E	7	M	2.135	PD	P	0.863	N	0.28
rs1216778719	Val187Met	N	-2.21	DG	0.038	PSD	0.725	N	-87	L	1.005	PB	P	0.783	N	0.22
rs1219649544	Arg346His	DL	-4.41	TL	0.109	PSD	0.528	N	-5	N	0.575	PB	P	0.791	N	0.17
rs1223729648	Tyr38His	DL	-4.29	DG	0.001	PD	1.000	E	74	M	3.29	PB	P	0.803	D	0.78
rs1230091567	Met134Ile	N	-1.14	TL	0.239	B	0.017	N	-62	L	1.935	PB	P	0.828	N	0.49
rs1235749501	His13Tyr	N	-0.16	TL	1.000	B	0.080	N	-74	N	0.6	PB	U	0.680	N	0.30
rs1240566715	Ser205Cys	N	-1.32	TL	0.272	B	0.010	N	-93	N	0.67	PB	U	0.706	N	0.38
rs1244026143	Val164Gly	DL	-6.56	DG	0.001	PD	0.998	E	75	H	4.825	PD	U	0.640	D	0.90
rs1244832277	Arg241Gln	DL	-2.96	DG	0.021	PSD	0.948	N	-9	L	1.225	PB	P	0.898	N	0.22
rs1247419183	Val337Met	DL	-2.93	DG	0.002	PD	1.000	E	58	M	2.105	PB	U	0.750	N	0.37
rs1268659696	Glu117Asp	N	-1.01	TL	0.610	B	0.000	N	-81	N	0	PB	U	0.587	N	0.24
rs1274533572	Gly327Val	DL	-8.79	DG	0.000	PD	1.000	E	62	M	2.745	PB	P	0.897	D	0.90
rs1276744835	Glu228Asp	DL	-2.76	TL	0.435	PD	0.999	N	-9	M	2.135	PB	U	0.760	N	0.07

rs1277434432	Met147Ile	N	-1.49	TL	0.164	B	0.005	N	-42	L	1.935	PB	P	0.746	N	0.11
rs1281415838	Ile103Val	N	-0.95	TL	0.066	B	0.151	N	-6	M	2.685	PB	U	0.676	N	0.33
rs1295342651	Gln404Arg	N	-1.24	TL	0.954	B	0.000	N	-79	N	0.49	PB	P	0.722	N	0.11
rs1295857274	Gln414Lys	N	-1.32	TL	0.268	B	0.000	N	-75	L	1.175	PB	U	0.645	N	0.30
rs1296782302	Pro93Arg	DL	-2.92	TL	0.71	B	0.436	N	-22	L	1.89	PB	P	0.865	D	0.82
rs1297345295	Val244Met	N	-2.20	TL	0.062	B	0.195	N	-67	L	1.055	PB	U	0.799	N	0.29
rs1298334491	Thr101Ile	N	-1.59	TL	0.135	B	0.000	N	-53	N	0.66	PB	U	0.626	N	0.31
rs1301434623	Asp44Ala	N	-2.12	TL	0.287	B	0.002	N	-47	N	-0.195	PB	P	0.844	N	0.32
rs1302148730	Leu384Phe	DL	-3.72	DG	0.001	PD	0.987	N	-10	M	2.115	PD	U	0.755	N	0.10
rs1308190883	Val416Met	N	-0.059	TL	0.102	PD	0.972	N	-85	M	2.075	PB	U	0.754	N	0.12
rs1310418829	Glu242Gly	DL	-5.55	DG	0.020	PD	0.965	N	-17	N	0.295	PB	U	0.720	D	0.88
rs1316268769	Pro70Ala	DL	-5.70	DG	0.012	PSD	0.925	N	-2	L	1.935	PB	U	0.727	D	0.56
rs1319030326	Glu132Lys	N	-0.95	TL	0.492	B	0.002	N	-37	L	1.935	PB	P	0.759	N	0.46
rs1324208253	Ser137Ala	N	-0.53	TL	0.332	B	0.000	N	-48	N	-0.145	PB	U	0.531	N	0.30
rs1335182846	Lys356Gln	N	-1.75	TL	0.250	B	0.142	N	-67	L	0.955	PB	U	0.725	N	0.26
rs1338237897	Ala5Gly	N	-0.94	TL	0.076	B	0.002	N	-90	L	1.04	PB	U	0.434	N	0.26
rs1338783398	Glu115Lys	N	-2.44	TL	0.054	B	0.022	N	-35	L	1.83	PB	P	0.796	D	0.57
rs1339827175	Glu242Lys	DL	-3.29	DG	0.004	B	0.068	N	-21	N	-0.245	PB	P	0.783	N	0.41
rs1340200537	Glu341Lys	DL	-3.89	DG	0.000	PD	1.000	E	91	M	2.75	PB	U	0.793	D	0.90
rs1340970650	Arg243Cys	DL	-7.13	DG	0.000	PSD	0.726	E	37	L	1.385	PB	P	0.878	N	0.44
rs1342613654	Lys400Arg	N	-2.07	TL	0.159	B	0.007	N	-74	L	1.12	PB	U	0.689	N	0.08
rs1352687393	Lys163Met	DL	-5.50	DG	0.000	PD	0.998	E	30	M	2.89	PB	U	0.712	D	0.90
rs1358103245	Glu49Gly	N	-2.17	N	0.353	B	0.157	N	-44	L	1.665	PB	P	0.799	N	0.43
rs1359340183	Glu365Lys	DL	-3.00	TL	0.150	B	0.154	N	-54	N	0.53	PB	P	0.800	N	0.18
rs1360782672	Met458Val	N	-1.31	N	0.169	B	0.000	N	-36	L	0.92	PB	U	0.641	N	0.36
rs1367895795	Ala230Thr	N	-1.56	DG	0.008	PD	0.993	N	-11	M	2.505	PB	P	0.846	N	0.24

rs1369639631	Phe425Leu	DL	-5.80	DG	0.001	PD	1.000	E	48	H	3.54	PB	P	0.874	D	0.89
rs1371607114	Thr146Ile	DL	-5.31	DG	0.002	PSD	0.917	E	22	L	1.935	PB	P	0.814	D	0.74
rs1373202839	Arg436Lys	DL	-2.53	TL	0.241	B	0.074	N	-58	M	2.25	PB	P	0.845	N	0.33
rs1373983688	Gly410Ala	N	0.36	TL	0.184	B	0.000	N	-21	L	1.045	PB	U	0.511	N	0.29
rs1377831067	Ala304Val	DL	-2.64	TL	0.223	B	0.399	N	-54	N	0.615	PB	P	0.804	N	0.19
rs1380514442	Lys20Glu	DL	-3.34	DG	0.001	PD	0.995	E	73	H	3.665	PB	P	0.812	D	0.67
rs1390179436	Val270Met	N	-2.00	DG	0.050	PD	0.991	E	25	L	1.88	PB	P	0.876	D	0.61
rs1390801321	Lys389Arg	N	-1.11	TL	0.283	B	0.005	N	-83	N	0.75	PB	U	0.675	N	0.24
rs1394191738	Arg391Gly	DL	-6.53	DG	0.001	PD	1.000	E	87	H	4.475	PD	P	0.834	D	0.90
rs1396308032	Arg67Gln	N	-2.38	TL	0.093	B	0.178	E	10	M	1.96	PB	P	0.866	D	0.62
rs1397852501	His265Tyr	DL	-5.61	DG	0.000	PD	0.999	E	13	H	3.61	PD	P	0.826	D	0.90
rs1404637346	Ile6Thr	DL	-2.68	DG	0.043	B	0.023	N	-53	M	2.555	PB	U	0.624	D	0.75
rs1418994529	Arg466Trp	DL	-3.65	DG	0.002	PD	0.997	E	38	M	2.085	PB	P	0.857	N	0.76
rs1424916218	Arg273Gln	DL	-3.45	DG	0.032	PD	0.999	E	81	H	3.635	PD	P	0.87	D	0.90
rs1431676094	Glu459Gly	N	0.11	TL	0.443	B	0.002	N	-55	N	-0.945	PB	P	0.747	N	0.28
rs1438024792	Glu355Gly	DL	-5.53	TL	0.053	PD	0.984	N	-19	L	1.61	PB	P	0.810	N	0.32
rs1444891733	Val412Met	N	0.004	DG	0.014	PSD	0.527	N	-34	L	1.83	PB	U	0.758	N	0.21
rs1445658903	Ala139Val	N	-0.064	TL	0.221	B	0.000	N	-83	N	0	PB	P	0.766	N	0.33
rs1454909926	Leu321Pro	DL	-6.61	DG	0.000	PD	1.000	E	49	H	3.72	PD	P	0.917	D	0.90
rs1462219795	Arg200His	DL	-3.88	TL	0.056	PSD	0.620	E	11	L	1.38	PB	U	0.662	N	0.21
rs1467510263	Ala476Val	N	-2.04	DG	0.039	PSD	0.953	N	-1	M	2.985	PB	P	0.801	D	0.85
rs1468001776	Tyr417Asp	DL	-4.73	TL	0.144	B	0.056	N	0	M	2.495	PB	P	0.860	D	0.69
rs1476861882	Leu52Phe	DL	-3.49	DG	0.003	PD	0.999	E	13	H	3.9	PB	P	0.776	D	0.62
rs1480573852	Lys377Arg	N	-0.67	TL	0.751	B	0.006	N	-82	L	1.3	PB	U	0.466	N	0.08
rs1566816661	Lys268Asn	N	-1.14	TL	0.333	PSD	0.867	N	-36	N	0.41	PB	U	0.693	N	0.17
rs1566818045	Ala139Pro	N	-0.74	TL	0.262	B	0.000	N	-85	N	0	PB	U	0.711	N	0.35

rs1566818890	Glu85Lys	DL	-3.53	DG	0.002	PD	1.000	E	41	M	2.06	PB	P	0.833	D	0.69
rs1566818959	Met63Ile	DL	-3.23	TL	0.260	B	0.009	N	-15	L	1.03	PB	P	0.873	D	0.51
rs1595239049	Thr448Pro	N	-2.26	TL	0.409	B	0.003	N	-77	M	2.74	PB	U	0.676	N	0.30
rs1595242014	Val337Gly	DL	-6.84	DG	0.0000	PD	1.000	E	47	M	2.975	PB	U	0.549	D	0.90
rs1595244672	Thr197Pro	DL	-5.34	DG	0.0042	PSD	0.736	N	-17	L	1.07	PB	U	0.725	D	0.89
rs1595245738	Glu116Lys	N	-1.60	TL	0.132	B	0.021	N	-48	L	1.83	PB	U	0.744	D	0.51
rs142646145	His89Gln	DL	-4.42	TL	0.142	PD	0.961	N	-34	L	1.675	PB	U	0.715	D	0.83
rs1892340313	Pro451Ser	DL	-7.38	DG	0.004	PD	1.000	E	44	M	3.205	PB	P	0.864	D	0.73
rs1892344387	Lys426Thr	DL	-4.97	DG	0.042	PD	0.969	N	-22	M	3.215	PB	P	0.842	N	0.48
rs1892477463	Ile366Thr	DL	-3.85	DG	0.010	PSD	0.471	N	-7	M	2.255	PB	U	0.753	D	0.79
rs1892486033	Arg328His	DL	-4.14	DG	0.040	PSD	0.916	N	-68	N	0.62	PB	U	0.801	N	0.17
rs1892478123	Met363Thr	DL	-2.90	TL	0.662	B	0.034	N	-81	N	-0.56	PB	P	0.863	N	0.26
rs1892503990	Met306Val	N	0.89	DG	0.004	PSD	0.810	E	20	N	0.23	PB	P	0.842	D	0.85
rs1892518022	Glu267Lys	N	-2.10	TL	0.168	B	0.001	N	-68	N	0.585	PB	P	0.882	D	0.54
rs1892518764	Ser266Ala	N	-1.41	TL	0.131	B	0.000	N	-49	L	1.655	PB	U	0.512	N	0.26
rs1892523027	Ala250Val	N	-1.35	TL	0.165	PSD	0.790	N	-81	L	1.25	PB	P	0.835	N	0.20
rs1892659749	Ser126Thr	N	-0.62	TL	0.232	B	0.002	N	-64	L	1.59	PB	P	0.749	N	0.33
rs1892954317	Arg23Gln	DL	-3.54	DG	0.002	PD	1.000	E	58	M	2.185	PB	P	0.860	N	0.46
rs1893685625	Ile6Val	N	-0.20	TL	0.770	B	0.000	N	-84	N	-0.235	PB	U	0.448	N	0.16
rs1264566007	Asn128Lys	N	-1.17	TL	0.272	B	0.019	N	-48	L	1.385	PB	P	0.869	N	0.46
rs1770844018	Lys307Glu	DL	-3.39	DG	0.009	B	0.409	E	45	N	0.765	PB	U	0.624	D	0.89
rs1883198727	Ser378Cys	DL	-3.97	DG	0.006	PD	0.995	N	-44	M	1.96	PB	U	0.732	D	0.59
rs1892308596	Ser477Asn	N	-1.28	DG	0.033	PSD	0.533	N	-19	M	2.91	PB	U	0.670	D	0.59
rs1892342047	Glu441Ala	DL	-5.53	DG	0.003	PSD	0.564	N	-29	M	3.015	PB	P	0.866	N	0.44
rs1892342488	Asp439Glu	DL	-3.50	DG	0.011	PD	0.993	N	-13	M	2.785	PB	U	0.778	N	0.46
rs1892447426	Gly395Ser	DL	-4.70	TL	0.107	PSD	0.938	N	-82	L	0.875	PB	P	0.822	D	0.63
rs1892447721	Gly394Arg	DL	-6.65	DG	0.002	PD	0.999	E	4	N	0.38	PB	P	0.890	D	0.72

rs1892472772	Lys385Arg	N	-0.97	TL	0.420	B	0.000	N	-95	N	0.675	PB	U	0.699	N	0.10
rs1892473652	Leu379Phe	DL	-3.80	TL	0.066	PD	0.999	N	-9	L	0.875	PD	U	0.713	N	0.19
rs1892475655	Thr371Ala	N	-2.35	TL	0.661	B	0.061	N	-59	N	0.445	PB	P	0.822	N	0.32
rs1892481082	Asp353Gly	DL	-6.69	DG	0.002	PD	0.996	E	59	L	1.78	PB	U	0.798	D	0.58
rs1892483378	Gly345Ser	DL	-5.48	DG	0.033	PD	0.992	E	30	M	2.91	PD	P	0.766	D	0.81
rs1892502921	Thr312Ile	DL	-5.53	DG	0.000	PD	0.984	E	82	H	3.595	PD	P	0.833	D	0.90
rs1892504590	Ile300Asn	DL	-6.63	DG	0.000	PD	0.998	E	28	N	0.47	PB	P	0.835	D	0.85
rs1892504858	Lys297Arg	DL	-2.89	DG	0.000	PD	1.000	E	6	N	0.72	PB	U	0.729	D	0.76
rs1892506436	Gly286Arg	DL	-7.21	DG	0.032	PD	0.999	E	82	M	2.27	PD	P	0.856	D	0.90
rs1892507073	Leu280Phe	DL	-3.76	DG	0.001	PD	0.996	E	47	N	0.385	PB	U	0.677	D	0.89
rs1892520149	Asp262Gly	N	-0.33	TL	1.000	B	0.021	N	-77	L	1.03	PB	U	0.735	N	0.23
rs1892520597	Leu261Pro	DL	-6.58	DG	0.000	PD	1.000	E	75	H	4.185	PB	P	0.871	D	0.90
rs1892525199	Arg243His	DL	-4.43	DG	0.049	PD	1.000	N	-42	L	0.96	PB	U	0.789	N	0.18
rs1892528134	Phe236Ser	DL	-7.34	DG	0.000	PD	1.000	E	67	L	1.51	PB	U	0.774	D	0.90
rs1892553720	Val226Leu	DL	-2.75	DG	0.002	PSD	0.951	E	63	L	1.375	PD	P	0.857	D	0.63
rs1892621826	Glu198Gly	DL	-6.50	DG	0.000	PD	1.000	E	63	M	3.25	PB	U	0.747	D	0.90
rs1892626748	Lys189Thr	DL	-5.31	DG	0.027	B	0.442	N	-10	L	1.73	PB	P	0.764	N	0.46
rs1892631024	Leu153Pro	DL	-6.17	DG	0.000	PD	0.980	E	61	M	3.165	PB	P	0.939	D	0.89
rs1892631592	Glu149Lys	N	-1.52	DG	0.026	B	0.022	E	29	N	0.69	PB	P	0.742	N	0.28
rs1892658092	Pro141Ser	N	-0.28	TL	0.190	B	0.000	N	-56	N	-<br>0.205	PB	U	0.711	N	0.43
rs1892658609	Val136Ala	N	-0.38	TL	0.745	B	0.002	N	-11	L	1.04	PB	U	0.726	N	0.48
rs1892659463	Ser129Leu	N	-1.28	TL	0.337	B	0.000	N	-32	L	1.355	PB	P	0.900	D	0.62
rs1892660002	Gly123Asp	N	-1.10	DG	0.045	B	0.005	E	3	L	1.7	PB	U	0.659	N	0.31
rs1892663954	Leu110Phe	DL	-3.45	DG	0.012	PD	0.957	E	14	L	1.935	PB	U	0.692	N	0.49
rs1892665030	Ala107Asp	DL	-3.44	DG	0.022	PSD	0.933	E	26	M	3.07	PB	P	0.856	N	0.45
rs1892666135	Ala102Val	DL	-3.48	TL	0.079	B	0.031	N	-23	M	2.49	PB	P	0.839	D	0.67
rs1892666993	Thr100Ile	N	0.48	TL	1.000	B	0.000	N	-72	N	-1.55	PB	P	0.799	N	0.31

rs1892732634	Glu94Lys	N	-1.10	TL	0.249	B	0.003	N	-43	L	1.54	PB	P	0.839	D	0.54
rs1892738131	Gln61Leu	DL	-6.20	DG	0.011	PSD	0.942	E	2	L	1.42	PB	P	0.816	D	0.83
rs1892948595	Ala58Val	N	-2.33	TL	0.216	PSD	0.940	N	-53	L	1.565	PB	P	0.762	D	0.50
rs1892948718	Ala58Ser	N	-1.11	TL	0.507	B	0.026	N	-71	L	1.46	PB	U	0.675	N	0.41
rs1892951094	Pro42Leu	DL	-8.29	TL	0.053	PSD	0.827	E	6	M	2.26	PB	P	0.798	D	0.65
rs1892954511	Arg23Trp	DL	-7.09	DG	0.000	PD	1.000	E	77	H	3.815	PB	P	0.845	D	0.84
rs1892954980	Thr21Ile	DL	-3.56	DG	0.004	PD	0.978	E	19	M	2.7	PB	P	0.818	D	0.64
rs1893686640	Ser2Asn	N	-0.27	TL	1.000	B	0.000	N	-99	N	0.41	PB	U	0.378	N	0.09
