# Supplementary material for: Discovering Deleterious Single Nucleotide Polymorphisms of Human AKT1 Oncogene: An In Silico Study
Source: Life (Basel). 2023 Jul 10;13(7):1532. doi: 10.3390/life13071532 (PMC10381612; doi:10.3390/life13071532)
Supplement: Supplementary file 1 [file life-13-01532-s001.zip › Supplementary Table S3.pdf]

**Supplementary Table S3:** Analysis of the effect of 3′ UTR SNPs on microRNA (miRNA) binding sites using PolymiRT analysis for gene AKT1. The functional classes "C" means the derived allele creates a new miRNA site, "D" means the derived allele disrupts a con-served miRNA site, "N" means the derived allele disrupts a nonconserved miRNA site, and "O" means it is not possible to determine the ancestral allele.

| dbSNP ID | Variant type | miR ID | miRSite | Function Class | context+ score change |
|---|---|---|---|---|---|
| rs190605178 | SNP | hsa-miR-3944-5p | ccGCTGCACcacg | O | -0.137 |
| | | hsa-miR-150-3p | ccgcTGTACCAcg | O | -0.073 |
| rs140320743 | SNP | hsa-miR-3944-5p | gctgccGCTGCAC | O | -0.137 |
| | | hsa-miR-143-5p | gctgccACTGCAC | O | -0.099 |
| rs183360200 | SNP | hsa-miR-3126-3p | GCCAGATgctgcc | O | -0.078 |
| rs3803305 | SNP | hsa-miR-1321 | ggcCCTCCCTgac | O | -0.089 |
| | | hsa-miR-149-3p | ggCCCTCCCtgac | O | -0.165 |
| | | hsa-miR-211-3p | ggcccTCCCTGAc | O | -0.08 |
| | | hsa-miR-4270 | ggccCTCCCTGAc | O | -0.3 |
| | | hsa-miR-4441 | ggccCTCCCTGAc | O | -0.212 |
| | | hsa-miR-4728-5p | ggCCCTCCCtgac | O | -0.137 |
| | | hsa-miR-4739 | ggcCCTCCCTgac | O | -0.099 |
| | | hsa-miR-4756-5p | ggcCCTCCCTgac | O | -0.071 |
| | | hsa-miR-6754-5p | ggccCTCCCTGAc | O | -0.223 |
| | | hsa-miR-6785-5p | ggCCCTCCCtgac | O | -0.118 |
| | | hsa-miR-6883-5p | ggCCCTCCCtgac | O | -0.118 |
| | | hsa-miR-3202 | ggCCCTTCCtgac | O | -0.088 |
| | | hsa-miR-4476 | ggcCCTTCCTgac | O | -0.055 |
| | | hsa-miR-6876-5p | ggcCCTTCCTgac | O | -0.055 |
| | | hsa-miR-873-5p | ggcccTTCCTGAc | O | -0.014 |
| rs112114610 | SNP | hsa-miR-7153-3p | tctCCATGGTAgc | C | -0.3 |
| rs1130443 | SNP | hsa-miR-3154 | cCCCTTCTgtgtc | D | -0.135 |
| | | hsa-miR-3179 | CCCCTTCtgtgtc | D | -0.155 |
| rs1130295 | SNP | hsa-miR-1915-3p | gggcCCCTGGGAt | D | -0.239 |
| | | hsa-miR-4726-3p | gggccCCTGGGAt | D | -0.077 |
| | | hsa-miR-6764-5p | gggcCCCTGGGAt | D | -0.239 |
| | | hsa-miR-6840-3p | gggccCCTGGGAt | D | -0.067 |
| | | hsa-miR-4733-5p | gggcccTTGGGAT | C | 0.026 |
| | | hsa-miR-6814-5p | gggcCCTTGGGAt | C | -0.175 |
| | | hsa-miR-7160-3p | GGGCCCTtgggat | C | -0.184 |
| rs1804268 | SNP | hsa-miR-6849-3p | tcggAGGCTGGgc | D | -0.026 |
| | | hsa-miR-1324 | gTGTCTGAggacg | O | 0.001 |
| rs34865723 | SNP | hsa-miR-1915-3p | agccgCCCTGGGc | D | -0.119 |
| | | hsa-miR-3141 | agCCGCCCTgggc | D | -0.194 |
| | | hsa-miR-4685-5p | agccGCCCTGGgc | D | -0.113 |
| | | hsa-miR-6764-5p | agccgCCCTGGGc | D | -0.119 |
| | | hsa-miR-6837-5p | agccGCCCTGGgc | D | -0.113 |
| | | hsa-miR-1825 | agccGCACTGGgc | C | -0.089 |
| rs137981458 | SNP | hsa-miR-1286 | gggcagGTCCTGC | D | -0.089 |
| | | hsa-miR-492 | gggCAGGTCCtgc | D | -0.09 |
| | | hsa-miR-18a-3p | GGGCAGAtcctgc | C | -0.103 |
| | | hsa-miR-1913 | GGGCAGAtcctgc | C | -0.107 |
| | | hsa-miR-324-3p | GGGCAGAtcctgc | C | -0.104 |
| rs61761249 | SNP | hsa-miR-1226-5p | tcaGCCCTCAgaa | D | -0.272 |
| | | hsa-miR-3616-3p | tcaGCCCTCAgaa | D | -0.154 |
| | | hsa-miR-4303 | tcagccCTCAGAA | D | -0.069 |
| | | hsa-miR-4446-3p | tCAGCCCTcagaa | D | -0.196 |

| | | | | | |
|---|---|---|---|---|---|
| | | hsa-miR-4649-3p<br>hsa-miR-4656<br>hsa-miR-4721<br>hsa-miR-7162-3p | tcagcCCTCAGAa<br>TCAGCCCtcagaa<br>tcAGCCCTCAgaa<br>tcagcCCTCAGAa | D<br>D<br>D<br>D | -0.087<br>-0.146<br>-0.36<br>-0.1 |
| | | hsa-miR-4256 | tcagccGTCAGAA | C | -0.086 |
| rs17846821 | SNP | hsa-miR-135a-5p<br>hsa-miR-135b-5p | AAGCCATgctgtc<br>AAGCCATgctgtc | C<br>C | -0.104<br>-0.104 |
| rs11555430 | SNP | hsa-miR-6089 | gaggCGGCCTCgt | D | -0.173 |
| | | hsa-miR-1281 | GAGGCGAcctcgt | C | -0.209 |
| rs192237287 | SNP | hsa-miR-6509-3p | ggcggCAGTGGAc | C | No Change |
