# Supplementary material for: Discovering Deleterious Single Nucleotide Polymorphisms of Human AKT1 Oncogene: An In Silico Study
Source: Life (Basel). 2023 Jul 10;13(7):1532. doi: 10.3390/life13071532 (PMC10381612; doi:10.3390/life13071532)
Supplement: Supplementary file 1 [file life-13-01532-s001.zip › Supplementary Table S4.pdf]

Supplementary Table S4: Information of AKT1 missense SNPs available from the Genomic Data Commons Data Portal (https://portal.gdc.cancer.gov/ accessed on 9 June 2023)

DNA change	Missense mutation	Number of affected cases in Cohort	Number of affected cases across GDC	Cancer type
chr14:g.104780214C>T	E17K	68 / 3,365	68 / 14783	29 cases of ductal and lobular neoplasms, 26 cases of adenomas and adenocarcinomas, 7 cases of squamous cell neoplasm, 3 cases of lymphoid leukaemia, 2 cases of nevi and melanomas, 1 case of transitional cell papilloma
chr14:g.104780145C>T	E40K	4 / 3,365	4 / 14783	2 cases of adenomas and adenocarcinomas, 1 case of ductal and lobular neoplasms, 1 case of thymic epithelial neoplasm
chr14:g.104776708A>G	W80R	4 / 3,365	4 / 14783	4 cases of adenomas and adenocarcinomas
chr14:g.104780108A>C	L52R	4 / 3,365	4 / 14783	2 cases of ductal and lobular neoplasms, 1 case of adenomas and adenocarcinomas, 1 case of squamous cell neoplasm
chr14:g.104776711G>T	Q79K	2 / 3,365	2 / 14783	1 case of adenomas and adenocarcinomas, 1 case of lymphoid leukaemia
chr14:g.104773083C>A	D323Y	2 / 3,365	2 / 14783	2 cases of adenomas and adenocarcinomas
chr14:g.104773013C>T	R346H	1 / 3,365	1/ 14783	1 case of cystic, mucinous and serous neoplasm
chr14:g.104775774T>C	T105A	1 / 3,365	1/ 14783	1 case of adenomas and adenocarcinomas
chr14:g.104775690C>T	E133K	1 / 3,365	1/ 14783	1 case of adenomas and adenocarcinomas
chr14:g.104770360G>A	S475L	1 / 3,365	1/ 14783	1 case of adenomas and adenocarcinomas
chr14:g.104780108A>T	L52H	1 / 3,365	1/ 14783	1 case of adenomas and adenocarcinomas
chr14:g.104773276C>T	G311D	1 / 3,365	1/ 14783	1 case of nevi and melanoma
chr14:g.104775696C>T	A131T	1 / 3,365	1/ 14783	1 case of adenomas and adenocarcinomas
chr14:g.104773086C>T	E322K	1 / 3,365	1/ 14783	1 case of squamous cell neoplasm
chr14:g.104770769T>C	I447V	1 / 3,365	1/ 14783	1 case of adenomas and adenocarcinomas
chr14:g.104774939G>A	T211I	1 / 3,365	1/ 14783	1 case of glioma
chr14:g.104770390C>T	R465H	1 / 3,365	1/ 14783	1 case of adenomas and adenocarcinomas
chr14:g.104776752G>A	T65M	1 / 3,365	1/ 14783	1 case of adenomas and adenocarcinomas
chr14:g.104775747C>G	E114Q	1 / 3,365	1/ 14783	1 case of adenomas and adenocarcinomas
chr14:g.104775693C>T	E132K	1 / 3,365	1/ 14783	1 case of transitional cell papilloma
chr14:g.104780118C>G	E49Q	1 / 3,365	1/ 14783	1 case of squamous cell neoplasm

chr14:g.104772373C>T	E418K	1 / 3,365	1/ 14783	1 case of adenomas and adenocarcinomas
chr14:g.104774970C>T	V201I	1 / 3,365	1/ 14783	1 case of glioma
chr14:g.104780120C>T	R48H	1 / 3,365	1/ 14783	1 case of adenomas and adenocarcinomas
chr14:g.104775726G>A	R121W	1 / 3,365	1/ 14783	1 case of adenomas and adenocarcinomas
chr14:g.104776693C>T	E85K	1 / 3,365	1/ 14783	1 case of epithelial neoplasm
chr14:g.104776741G>A	R69W	1 / 3,365	1/ 14783	1 case of squamous cell neoplasm
chr14:g.104773085T>C	E322G	1 / 3,365	1/ 14783	1 case of adenomas and adenocarcinomas
chr14:g.104780165C>T	G33D	1 / 3,365	1/ 14783	1 case of squamous cell neoplasm
chr14:g.104772900G>A	L384F	1 / 3,365	1/ 14783	1 case of cystic, mucinous and serous neoplasm
chr14:g.104773354T>A	D285V	1 / 3,365	1/ 14783	1 case of squamous cell neoplasm
chr14:g.104770816G>A	S431L	1 / 3,365	1/ 14783	1 case of adenomas and adenocarcinomas
chr14:g.104773083C>T	D323N	1 / 3,365	1/ 14783	1 case of cystic, mucinous and serous neoplasm
chr14:g.104780133C>T	D44N	1 / 3,365	1/ 14783	1 case of adenomas and adenocarcinomas
chr14:g.104774991G>A	H194Y	1 / 3,365	1/ 14783	1 case of nevi and melanoma
chr14:g.104776660G>A	R96W	1 / 3,365	1/ 14783	1 case of adenomas and adenocarcinomas
chr14:g.104780106T>G	N53H	1 / 3,365	1/ 14783	1 case of adenomas and adenocarcinomas
chr14:g.104770841G>T	P423T	1 / 3,365	1/ 14783	1 case of cystic, mucinous and serous neoplasm
chr14:g.104775741C>T	E116K	1 / 3,365	1/ 14783	1 case of transitional cell papilloma
chr14:g.104775122C>T	R174H	1 / 3,365	1/ 14783	1 case of adenomas and adenocarcinomas
chr14:g.104780118C>T	E49K	1 / 3,365	1/ 14783	1 case of squamous cell neoplasm
chr14:g.104773050C>A	G334W	1 / 3,365	1/ 14783	1 case of transitional cell papilloma
chr14:g.104792634C>G	V4L	1 / 3,365	1/ 14783	1 case of ductal and lobular neoplasm
chr14:g.104776710T>C	Q79R	1 / 3,365	1/ 14783	1 case of glioma
chr14:g.104770769T>A	I447F	1 / 3,365	1/ 14783	1 case of cystic, mucinous and serous neoplasm
chr14:g.104792600C>T	R15Q	1 / 3,365	1/ 14783	1 case of squamous cell neoplasm
chr14:g.104775688C>G	E133D	1 / 3,365	1/ 14783	1 case of adenomas and adenocarcinomas
chr14:g.104780189C>T	R25H	1 / 3,365	1/ 14783	1 case of complex epithelial neoplasm
chr14:g.104772942G>A	R370C	1 / 3,365	1/ 14783	1 case of complex epithelial neoplasm
chr14:g.104776669G>A	P93S	1 / 3,365	1/ 14783	1 case of nevi and melanoma
